# Implications of seismic and GNSS strain rates in Himachal, Kashmir and Ladakh

**DOI:** 10.1038/s41598-023-48997-3

**Published:** 2023-12-08

**Authors:** T. S. Shrungeshwara, Bhavani Narukula, Sridevi Jade, Sapna Ghavri, Chiranjeevi G. Vivek, I. A. Parvez

**Affiliations:** 1 CSIR-4PI, CSIR Fourth Paradigm Institute (Formerly CSIR-CMMACS), Wind Tunnel Road, Bangalore, 560037 India; 2 Indian National Centre for Ocean Information Services (INCOIS), Ocean Valley, JNTU Road, Nizampet, 500090 Hyderabad India

**Keywords:** Solid Earth sciences, Seismology, Tectonics

## Abstract

We report the present day GNSS velocities (2015–2021) and strain rates in Himachal, Kashmir and Ladakh Himalaya covering the rupture zones of the 2005 Muzaffarabad earthquake and the 1905 Kangra earthquake. Geodetic strain rates estimated from GPS velocities of about 58 sites spanning last two decades of measurements indicate a mean compression rate of − 32.5 ns/year (nanostrain/year) and dilatation of − 37.3 ns/year. Seismic strain rates are estimated using both the instrumental period (1964–2021) and historical earthquakes since 1500 AD in this region. Seismic strain rates during the instrumental period of the past 50 + years indicate a mean compression rate of − 28.1 ns/year and it slightly decreases to − 21.7 ns/year after including the historical earthquakes of the past 520 years. The Azimuth of the seismic strain tensor for the instrumental and historic periods and geodetic strain tensor is broadly consistent with orientation of major faulting in this region suggesting uniform compression over a long-time interval justifying combined analysis of the strain rate field to determine the seismic potential of the region. Composite analysis of geodetic and seismic strain rates and the associated moments estimate the accumulated strain budget of ~ 1E + 21 Nm in the past 520 years which has a potential of generating future earthquake of M_w_ > 8 in this segment of Northwest Himalaya.

## Introduction

**Figure 1:**
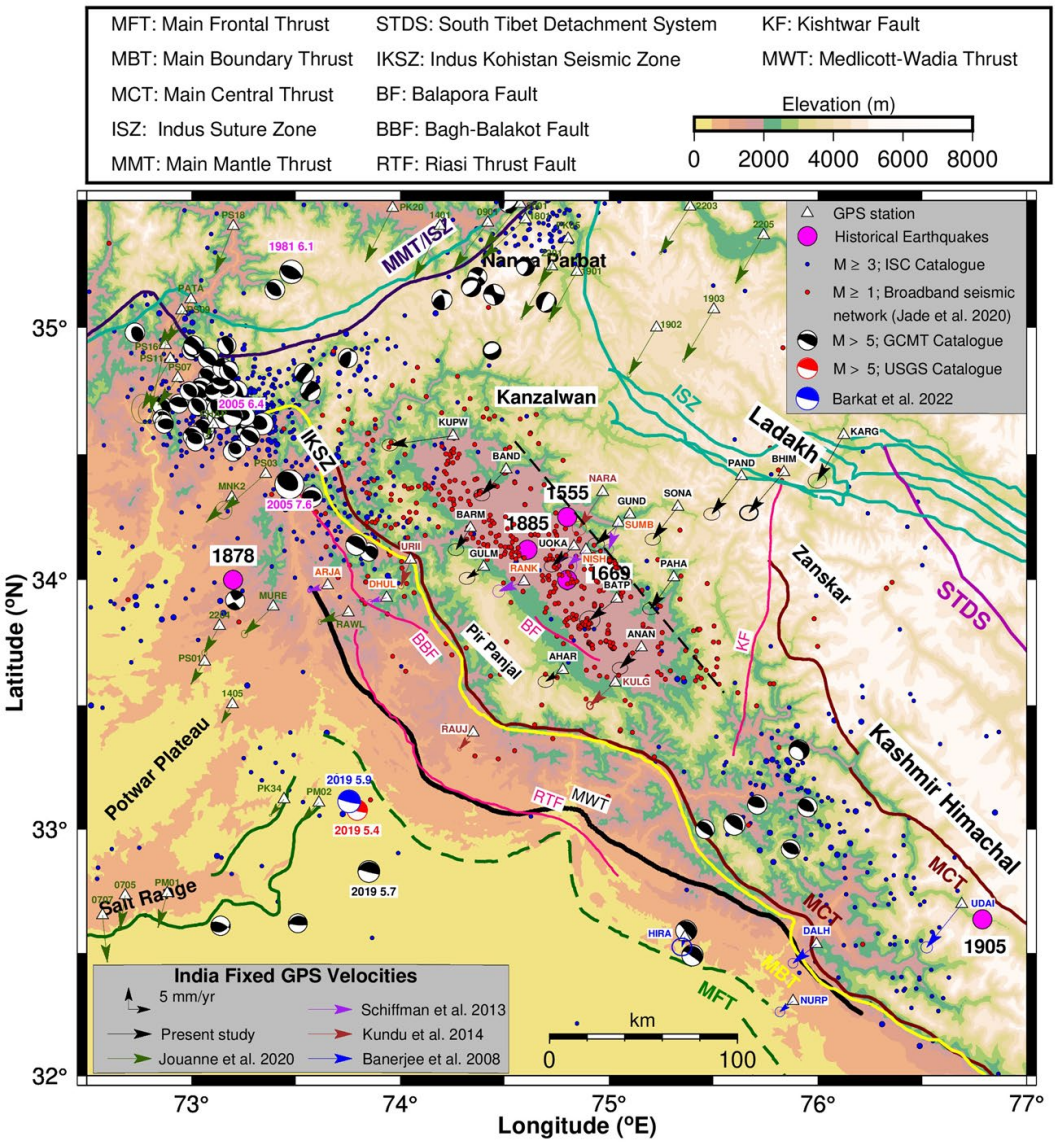
Map of the study region along with major faults/thrust lines mapped using^10,16,40,53–57^. Black dashed line is the northern edge of locked decollement estimated using data from collocated broadband seismic network^16^. Figure was created using GMT (Generic Mapping Tool) software version 6.0.0^58^.

Current study region (32°–35.5° N; 72.5°–77° E) covering Kashmir, Ladakh and Himachal Himalaya is tectonically complex and seismically active as it includes the rupture zones of the M_w_ 7.6, 2005 Kashmir (Muzaffarabad) earthquake and M_w_ 7.9, 1905 Kangra earthquake (Fig. 1). This region experienced about 20 major to moderate earthquakes dating back to the ninth century^1,2^ though the accurate details of these events have large discrepancies in timing and intensity. Notable reasonably documented historical earthquakes in this region: 1555 Kashmir earthquake of M_w_ 7.6–8.2^3–10^, the 1669 Srinagar earthquake of M_w_ 6.5–7.0^9^, the 1878 Abbottabad earthquake of M_w_ 6.76^4^, the 1885 Srinagar earthquake of M_w_ 6.4^5,11^ and the 1905 Kangra earthquake of M_w_ 7.8–8.0^4,5,8,12^. In addition, other significant earthquakes from historical records in the study region are 1501 M_w_ 6.5–7, 1678 M_w_ 6.5–6.8, 1683 M_w_ 6.5–6.8, 1736 M_w_ 6.5–7, 1779 M_w_ 6.5–7.5, 1784 M_w_ 6.5–7.5, 1828 M_w_ 6.5–7.5 and 1863 M_w_ 6 with epicentral coordinates estimated from felt locations^9^. Prior to 1500 two earthquakes of M_w_ 6.5–7.5 occurred in the year 844 and 1123 which do not have sufficient historical records^9^.

According to historical data records (Table 2), strong shaking was documented during 4 April, 1905 Kangra earthquake and the 1555 Kashmir earthquake (Fig. 1). The 1905 Kangra earthquake with a focal depth of 15 km was the most damaging earthquake that is located to the Southeast of Kashmir valley^9^. It may be noted that about 20,000 human lives were lost due to this earthquake^4,5,7,8,13^. The 1878 Abbottabad earthquake of M_w_ 6.76^4,7^ caused damage at Abbottabad, Kohat, Peshawar, Attock, Rawalpindi and was strongly felt at Jhelum, Murree, Shimla, Mussoorie. Focal mechanisms of earthquakes with magnitude ≥ 5 during the instrumental period from 1964 to till date are plotted in Fig. 1 (https://www.globalcmt.org/CMTsearch.html). Seismic events with magnitude ≥ 3 with an epicentral error of less than 5 km are plotted in Fig. 1 from International Seismological Centre (ISC, www.isc.ac.uk/iscbulletin/search/catalogue, last access: March 2022) revised catalogue^14^. In addition, events of M_w_ ≥ 1 with less than 5 km epicentral error are plotted from our broadband seismic observation network^15,16^. Seismicity clusters indicate that this region is currently experiencing high concentration of seismic strain.

Major geological/tectonic features such as faults, thrusts, etc. at the tectonic plate boundaries are caused due to tectonic forces related to the movement of plates. Active deformation along these tectonic features are the major causes of earthquakes. The Indian tectonic plate subducts under the Eurasian plate along a subsurface thrust at a depth of 15–20 km and at a distance of 70–100 km from frontal Himalaya. This thrust is termed as Main Himalayan thrust (MHT). Surface expressions of the major thrusts (Fig. 1) in the Himalayan arc from south to north are Main Frontal Thrust (MFT), Main Boundary Thrust (MBT), Main Central Thrust (MCT), South Tibet Detachment System (STDS) and Indus Suture Zone (ISZ) which demarcates the Himalayas into frontal, lesser, higher, tethyan and trans Himalayan regions. Surface expression of MHT merges with the MFT. The tectonic features to the west in Pakistan (Fig. 1) are Main Mantle Thrust (MMT), Indus Kohistan Seismic Zone (IKSZ), and Northwest-Southeast trending Bagh-Balakot Fault (BBF) from Indus to Jhelum valley. Regional fault systems in this region caused by seismic forces are Riasi Thrust Fault (RTF) located in the frontal Himalaya to the south of Pir Panjal ranges, northwest-southeast trending Balapora Fault (BF) located in the southeast region of Kashmir valley, Kishtwar Fault (KF) running from south to north from Kishtwar to Zanskar ranges and Karakoram fault in Ladakh Himalaya which extends further east of our study region to western Nepal system.

The October 8, 2005 Kashmir earthquake in IKSZ along the Balakot-Bagh Thrust (BBT), located in Muzaffarabad occurred during instrumental period and hence its epicentre, magnitude (M_w_ 7.6), and rupture zone are well constrained. This earthquake caused severe damage and took at least 80,000 lives in Northern Pakistan and Kashmir^10,17–21^. This earthquake ruptured an out of sequence Himalayan thrust (Fig. 1) known as Balakot-Bagh thrust located above the gently dipping Main Himalayan Thrust (MHT), at a distance of 150 km northeast of the Main Frontal Thrust. This earthquake was studied in detail by several researchers with different techniques resulting in well-constrained rupture zone, slip distribution, co-seismic slip and post-seismic deformation^18,22^. Studies indicate that the region within the 100 km radius of the rupture zone of the 2005 earthquake is currently experiencing post-seismic relaxation. Previous studies^1,2,5^ indicate that historical 1885 earthquake and 2005 Muzaffarabad event were approximately contiguous and may have occurred (http://cires1.colorado.edu/~bilham/Kashmir%202005.htm) on the same 32°–35.5° northeast dipping ramp. Mirpur earthquake occurred on 24 September 2019 with M_w_ 5.4 and 5.7 with depth of 11.5 and 14.7 km near MFT as per USGS and GCMT catalogue respectively (Figs. 1 and 4). Detailed study of Mirpur earthquake by integrating geodetic, seismic and field observation indicate a shallow depth of 6 km rupture and M_w_ of 5.9 (Figs. 1 and 4)^23^.

GPS studies in this region were initiated in 1995 across the Kangra rupture, 1997 in Ladakh region, after 2005 in the rupture zone of Muzzaffarabad earthquake and since 2008 in Kashmir valley and adjoining regions^10,16,19,20,24–31^. These studies gave significant insights into the active tectonics and geodetic surface deformation in this region. GPS measurements gave an arc-normal convergence rate of 14–16 mm/year and arc-parallel extension rate of 7–9 mm/year pointing to oblique deformation in this region^16^. Inverse models of surface deformation give an oblique slip rate of 14–16 mm/year along MHT at a depth of ~ 15 km with a locked width of 100–150 km^16^. Episodic GPS measurements made in Salt Range (Fig. 1) from 2007 to 2019^10^ record southward velocities suggesting weak coupling between the Salt Range and basal thrust of Potwar Plateau (Figs. 1 and 4), pointing to existence of massive salt layer. Further, GPS velocities suggest a southward horizontal flux in the central part of the front salt range. GPS measurements following the 2005 Muzaffarabad earthquake gave precise estimates of post-seismic deformation and associated slip models^16^. It was estimated that Balakot-Bagh thrust absorbs about 3 mm/year of shortening based on dating of previous events and co-seismic slip of 2005 Muzaffarabad event^10^. These studies indicate, the after-slip seismic moment of about 56 ± 19% of the seismic moment released by the main shock and the characteristic relaxation time is ~ 8.8 years^20^. Geodetic strain rates, dislocation models and micro-seismicity in Kashmir seismic gap suggest high strain accumulation to the north of Kashmir valley and south of Zanskar ranges pointing to a probable future large earthquake of M_w_ 7.7 in this region^16^.

Strain budget of the study region is crucial to address the seismic potential of the region, hence it is essential to carry out the comprehensive analysis of both geodetic strain rates determined from GNSS measurements and seismic strain rates determined from earthquake catalogues. With this objective, we established collocated continuous GNSS and Broadband seismic network in Kashmir Valley and adjoining regions in 2012 to study the ongoing deformation, micro seismicity and crustal structure. In this study we use about two years of new data till 2021 in addition to the earlier published data^16^ to determine the decadal crustal velocities. Comprehensive geodetic strain rates are estimated using velocities of about 58 GPS sites with a good spatial spread and long span of data in this region which includes our network and the published results of this region till date. Historical and current seismicity data in this region is used to determine the seismic strain rates. Further, composite analysis of seismic and geodetic strain rates is carried out to estimate the strain budget in this region which would give an indication of the possible occurrence of future earthquakes and their recurrence interval.

## Results and discussions

### GPS displacements

Both ITRF14 and Indian Plate reference frame velocities and associated uncertainties of the cGPS sites (Table 1) of the study region are estimated using the methodology described in Data and Methods section. India fixed velocities of campaign and cGPS sites (Fig. 1) obtained from our analysis and the published velocities^10,29–31^ are used for strain computations. The arc-normal and arc-parallel velocities of all the 58 GPS sites are determined by rotating the site velocities to the local arc geometry as defined by^32^ and plotted in Fig. S2a,b. Arc normal rates in this region indicate a surface convergence rate of 5–14 mm/year from the lesser to the Tethyan Himalaya suggesting predominantly high compression. This new data confirms our earlier hypothesis^16^ that the Balapora fault is currently active with convergence rate of about 3 mm/year and Kupwara located to the extreme northwest of Kashmir valley is recording about 9 mm/year westward velocity relative to Kashmir valley.

**Table 1:** ITRF 14 and India fixed rates of cGPS sites of our network with location description and the data span.

Site code	Lat (°N)	Lon (°E)	Epoch	ITRF14 velocities (mm/year)				India fixed velocities (mm/year)				Description
				N	σN	E	σE	N	σN	E	σE	
Kashmir/Ladakh Himalayan sites												
Kupwara KUPW	34.6	74.3	2015–2021	32.41	0.93	18.10	0.95	− 2.08	0.95	− 14.40	1.24	North-western part of Kashmir Valley
Baramulla BARM	34.2	74.3	2015–2021	29.56	0.92	29.41	0.94	− 4.94	0.94	− 3.31	1.23	Located on the bank of Jhelum River, also near NW edge of the valley
Gulmarg GULM	34.1	74.4	2015–2021	31.91	0.93	29.04	0.95	− 2.60	0.95	− 3.79	1.24	Located on foothills of Pir Panjal Range SE of Baramulla
Bandipora BAND	34.4	74.5	2015–2021	28.78	0.96	27.11	0.98	− 5.74	0.97	− 5.55	1.27	Located on the NW of Wular Lake
Aharbal AHAR	33.6	74.8	2015–2021	31.89	0.92	29.27	0.94	− 2.66	0.94	− 3.90	1.23	Southwestern part of Kashmir valley, on foothills of Pir Panjal
Srinagar UOKA	34.1	74.8	2015–2021	30.11	0.96	27.95	0.97	− 4.45	0.97	− 4.98	1.26	Located in the centre of Kashmir valley, on western bank of Dal Lake
Batpal BATP	33.9	75.0	2015–2017	29.73	1.41	26.96	1.41	− 4.85	1.42	− 6.15	1.62	NE edge of the valley, NNW of ANAN
Gund GUND	34.3	75.1	2015–2019	27.74	1.04	25.26	1.05	− 6.85	1.05	− 7.69	1.32	Located on bank of Sind river
Anantnag ANAN	33.7	75.2	2015–2021	29.83	0.92	28.37	0.94	− 4.77	0.94	− 4.88	1.23	South-eastern edge of the basin
Pahalgam PAHA	34.0	75.3	2015–2021	27.52	0.93	27.88	0.95	− 7.09	0.95	− 5.27	1.24	Located near Lidder river, Tethys Himalaya
Sonamarg SONA	34.3	75.3	2015–2021	27.19	0.92	27.28	0.94	− 7.43	0.94	− 5.73	1.23	Located on the bank of Sind river, NE of the valley
Pandrass PAND	34.4	75.6	2015–2021	26.02	0.96	26.30	0.98	− 8.63	0.97	− 6.75	1.27	Indus suture zone, Zanskar
Bhimbat BHIM	34.4	75.8	2017–2021	25.37	1.16	25.14	1.18	− 9.30	1.17	− 7.97	1.43	Indus suture zone, Zanskar
Kargil KARG	34.6	76.1	2017–2021	24.24	1.18	27.13	1.20	− 10.46	1.19	− 6.00	1.45	Indus suture zone, Zanskar

GPS measurements made for a period of 4–5 years within 100 km radius of the 2005 earthquake estimate post seismic displacement of about 10–60 mm/year soon after the earthquake and reducing through time in the hanging wall of BBT^20,21,31^. High post seismic displacement values are observed at the stations located within the 50 km radius of the epicentre. Our observation network has four GPS sites (KUPW, BAND, BARM, GULM) located beyond 50 km and within 100 km radius of 2005 event (Table 1 and Fig. 1). However only Kupwara site located at a radial distance of ~ 75 km to the extreme northwest of the valley recorded high westward velocity 14 mm/year i.e. ~ 9 mm/year relative to the rest of the valley sites indicating a component of post-seismic displacement related to 2005 Muzaffarabad earthquake. Given that our GPS measurements at Kupwara are from 2015 to 2021 i.e. covering 10–16 years post the 2005 event, Kupwara velocity suggests that this region may be recording the post-seismic deformation even 16 years after the 2005 event. However, we cannot rule out the possibility that Kupwara velocity may have a component contributed by active deep-rooted fault in Kanzalwan^16^. GPS measurements in Nangaparbhat further north of Kanzalwan^10^ suggest existence of an active thrust along an inverted fault plunging eastward in this region.

### Geodetic strain rates

**Figure 2:**
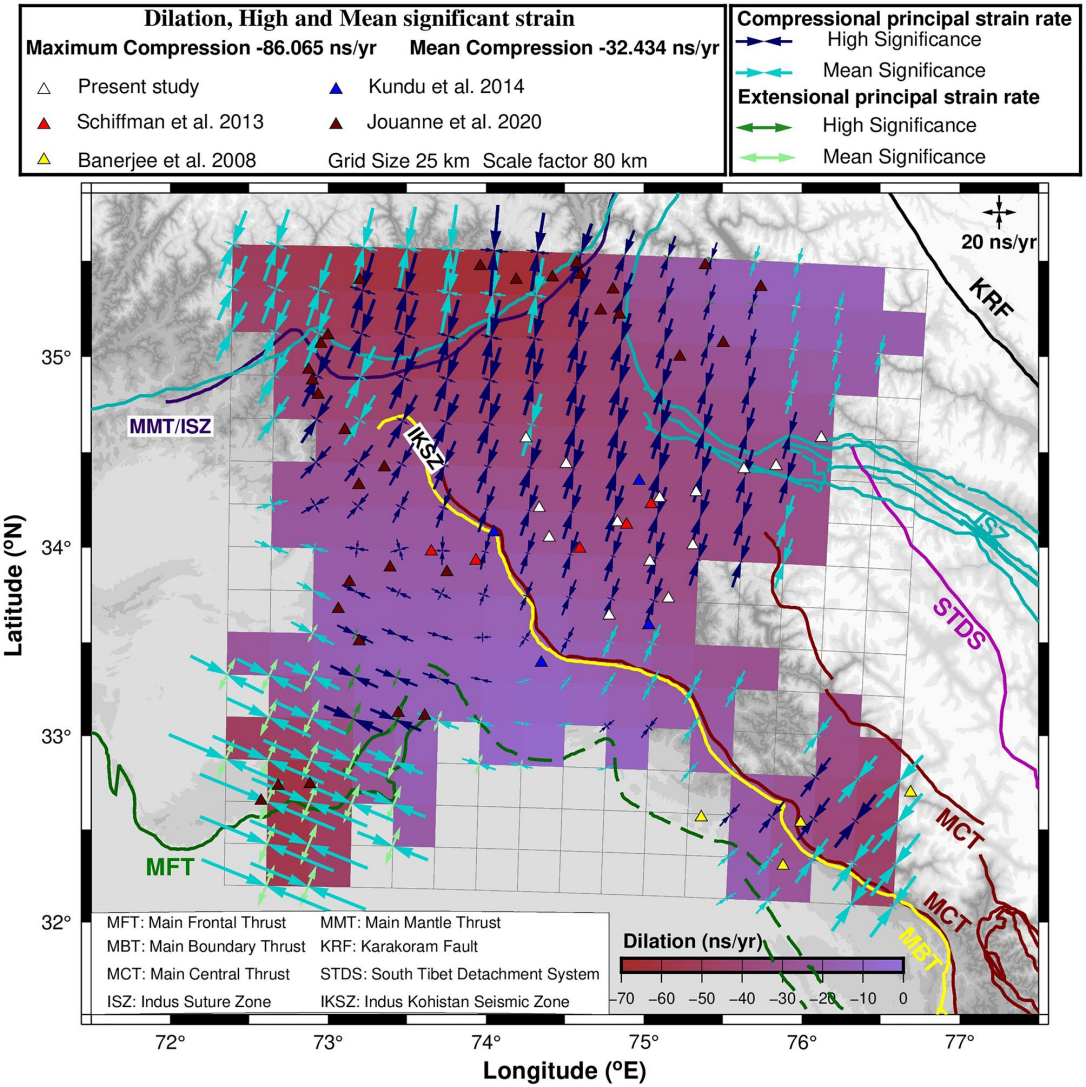
Geodetic strain rates and dilatation of study region. Major faults/thrust lines mapped using^10,16,53–57^. GPS site locations are denoted by solid triangles. The Figure was created using GMT (Generic Mapping Tool) software version 6.0.0^58^.

Our 14 cGPS site velocities (Table 1) along with published velocities of 44 additional GPS sites i.e., 4 stations^29^, 5 stations^30^, 4 stations^31^ and 31 stations^10^ (Table S1) are used for strain computation. Out of total 58 GPS sites with uncertainty limit less than 3 mm/year, there are 33 sites with < 1 mm/year uncertainty, 21 sites with 1–2 mm/year uncertainty and 4 sites with 2–3 mm/year uncertainty. We chose 25 km × 25 km grid size and scale factor of 80 km to compute strain rates. High and mean significant strain rates determined for Kashmir, Ladakh, Himachal and adjoining regions are plotted in Fig. 2 and listed in Table S3.

The maximum and minimum principal strain rates of high significance vary from − 16.1 to 22.4 ns/year and − 53.9 to − 9.7 ns/year with a mean value of − 2.7 and − 29.6 ns/year respectively. The maximum and minimum principal strain rates of mean significance vary from − 16.9 to 26.5 ns/year and − 86.1 to − 8.0 ns/year with a mean value of 2.6 and − 35.9 ns/year. The orientation of the minimum principal strain axes ranges between N 1.5° to N 165° with mean orientation angle of ~ 45° N. Dilatation of the study region ranges between − 70.0 to − 6.3 ns/year. The compression rates, with a mean − 32.4 ± 7 ns/year, are higher than extension rates indicating high compression in this region. Further, large negative value of dilatation (Fig. 2) substantiate that the study region is under high compression with mean dilatation rate of − 32.8 ns/year.

Extension rates observed in the Potwar Plateau and Salt Ranges (Fig. 2) are due to the existence of decollement between the two in the form of massive salt layer and horizontal southward flow in this layer^10^. Direction of compression strain in this region is manifestation of southward GPS velocities of the sites in Salt Range and is perpendicular to the direction of the thrusting of the Mirpur event to the east. Hence, we used the geodetic strain rates computed by excluding the three GPS points (0705, 0707, PM01) in Salt Range for estimating the seismic potential of the study region. The maximum and minimum compression rates are − 58.1 and − 16.0 ns/year with a mean compression rate of − 32.5 ns/year (Fig. S3) with predominant orientation angle of 24° N.

Geodetic strain rates contain both seismic and aseismic strain accumulation and hence provide reliable constraints on earthquake occurrence rate estimates for large enough regions. However, the GPS data should sample large spatial scale so as to minimise the non-linear strain accumulation during the earthquake cycle on individual faults. Further, the data span of geodetic measurements should be long enough to reduce the uncertainties on the estimated velocities. Our data analysis satisfies both these conditions and the mean geodetic strain rate of − 0.32 × 10^–7^ year^−1^ is sufficient for meaningful comparison of average strain rates^33^. Previous studies^34^ suggest that earthquake catalogues of 200–300 years are sufficient enough to determine the recurrence interval of earthquakes for regions straining at 10^–7^ year^−1^.

### Seismic strain rates

We need proper accurate data of magnitude and frequency information of past earthquakes, to determine the return periods reliably. Magnitudes and rupture zones of Historical earthquakes are (http://cires1.colorado.edu/~bilham/Kashmir%202005.htm) associated with large uncertainties (Table 2) due to non-availability of proper scientific records of historical earthquakes. Earthquake catalogues with well documented seismic events are available since 1964 and seismic events before that period are considered to be historical earthquakes.

**Table 2:** Historical major to great earthquakes in the study region (Reasonably documented earthquakes are marked in bold).

Date	Lon (°E)	Lat (°N)	Magnitude (M_w_)	Preferred (M_w_)	References
844	74.8	34	6.5–7.5	7.0	^9^
1123	74.8	34	6.5–7.5	7.0	^9^
1501-09-24	74.8	34	6.5–7.0	6.75	^9^
**1555-09**	**74.8**	**34.25**	**7.6**–**8.2**	**8.0**	^9,10,40^
**1669-06-23**	**74.8**	**34**	**6.5**–**7.0**	**7.0**	^9^
1678	74.8	34	6.5–6.8	6.65	^9^
1683	74.8	34	6.5–6.8	6.65	^9^
1736	74.8	34	6.5–7.0	6.75	^9^
1779	74.8	34	6.5–7.5	7.0	^9^
1784	74.8	34	6.5–7.5	7.0	^9^
1828	74.8	34	6.5–7.5	7.0	^9^
1863	74.8	34	6.0	6.0	^9,11^
**1878-03-02**	**73.2**	**34**	**6.76**	**6.76**	^4^
**1885-05-30**	**74.6**	**34.1**	**6.4**	**6.4**	^5,7^
**1905-04-04**	**76.8**	**32.6**	**7.8–8.0**	**7.9**	^4,5,8,9^

#### Instrumentation period

**Figure 3:**
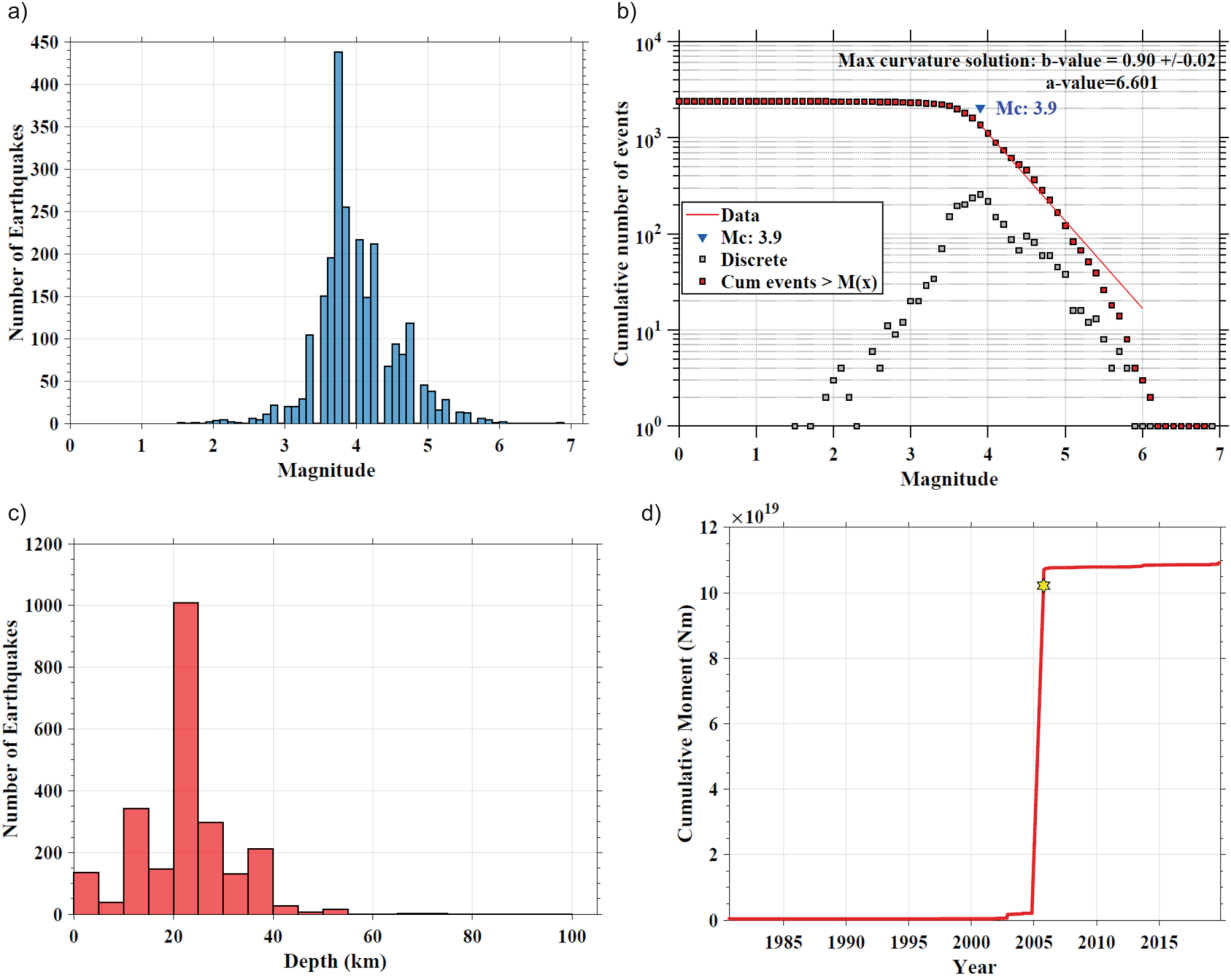
Characteristics of seismicity from 1964 to 2021 in study region. (**a**) Frequency plot of different size of events gives the magnitude completeness value of 3.9. (**b**) b-value estimation using magnitude completeness from maximum curvature method. The squares and triangles represent the cumulative and non-cumulative number of different size of events respectively. (**c**) Depth wise frequency of events indicate maximum frequency at 20 km hypocentral depth. (**d**) The cumulative measure of scalar seismic moment of the earthquake with time.

**Figure 4:**
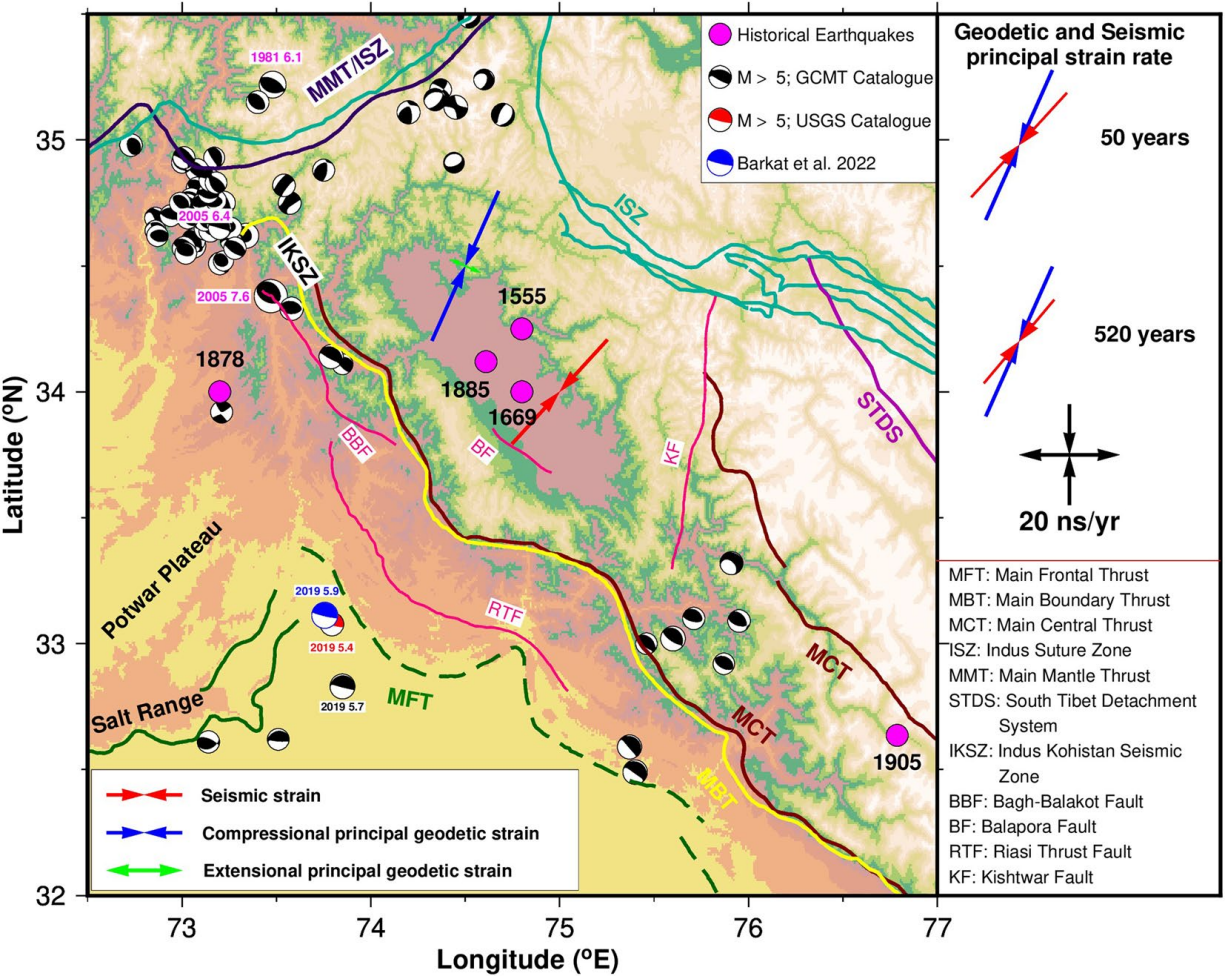
Focal mechanism of seismic events of magnitude > 5 are plotted from Global CMT catalogue (https://www.globalcmt.org/CMTsearch.html) with black color. Major faults/thrust lines mapped using^10,16,53–57^. The Figure was created using GMT (Generic Mapping Tool) software version 6.0.0^58^.

Using ZMAP^35^, we calculated the magnitude completeness [M_c_] and seismogenic thickness of the crust in the region based on the reported events from 1964 to 2021 from ISC catalogue (www.isc.ac.uk/iscbulletin/search/catalogue, last access: March 2022)^14^. Magnitude-Frequency distribution curve (Fig. 3a), total release of cumulative seismic moment (Fig. 3d) and Maximum likelihood solution (Fig. 3b) of the events during instrumentational period estimate Magnitude completeness M_c_ of the catalogue as 3.9. Hence, we estimated principal seismic strain rates in the region using available focal mechanism solutions of earthquakes M_w_ ≥ 3.9 reported in the GCMT catalogue (Table S2). The average seismogenic depth of the crust is taken as 20 km (Fig. 3c) for seismic strain analysis of 499.5 km (length) × 388.5 km (width) zone of the study area. 1964–2021 catalogue of about 50 + years contains the 2005 Kashmir earthquake (M_w_ 7.6), three strong earthquakes (6 ≤ M_w_ < 7), 46 moderate earthquakes (M_w_ 5–M_w_ 6), and several small to minor earthquakes. Using the empirical relations (Eqs. 7–13) provided by^36,37^, moment tensors were computed using strike, dip, and rake of the earthquakes listed in Table S2. Further principal seismic strain rates are calculated using (Eq. 6)^38^ formulation technique. The seismic strain deduced from 50 + years of data is − 28.1 ns/year with orientation of N 43° (Table 3; Fig. 4).

**Table 3:** Geodetic and Seismic strain rates.

Data	Data Span	Principal compressive strain rate (ns/year)	Azimuth
Geodetic	26 years (1996–2021)	− 32.5	24°N
Seismicity	Instrumental period	− 28.1	43°N
	Historical period	− 21.7	40°N

Mean Geodetic strain rate estimated from GPS velocities is − 32.5 ± 7 ns/year with orientation angle of ~ 24° N is higher than the seismic strain rate of − 28.1 ns/year with orientation of 43° N. Strain axes orientation of NNE are consistent with the orientation of major active faults in the region and the difference of about 19° N in the orientation angle suggest complex active tectonic regime in this region. Considering that the geodetic strain rate is the stored strain energy and the seismic strain rate is the strain release due to earthquakes, higher geodetic strain rate suggest that accumulated strain is not completely released by the earthquakes. 2005 Muzaffarabad earthquake is the major seismic event during this 50 + year period which caused mean co-seismic shortening of ~ 4.3 m indicating only partial release of accumulated strain of 200–300 years and suggesting that the remaining strain may be released by future earthquake on an active fault elsewhere or alternatively it may be accommodated by aseismic slip on the Salt Range Thrust^18^. Post seismic displacements^10,20^ support the hypothesis of after slip along a flat north of the ramp of main event rather than the hypothesis of viscous relaxation of lower crust. Afterslip hypothesis was also confirmed by^39^ using space geodetic observations and post seismic deformation models. Further lower seismic strain also indicates that the length of the catalogue is too short compared to the recurrence interval of earthquakes.

#### Historical period

Historical seismicity of the study region based on available record indicates 15 damaging earthquakes since the year 844, though the accurate magnitude and epicentre of these earthquakes could not be determined due to lack of data^9^. Paleo-seismic studies in the Riasi thrust section of MWT (Medlicott Wadia Thrust) located in the 200 km long seismic gap between 1905 Kangra earthquake M_w_ 7.8 and 2005 Balakot-Bagh M_w_ 7.6 indicate 500–700 years recurrence interval of large seismic events^40^. Seismic coupling study in northwest Himalaya (31.5–37° N; 71–77° E) indicate a recurrence interval of 500 years for M_w_ 8.51, 1000 years for M_w_ 8.62 and 2000 years for M_w_ 8.76 in this region^10^.

We assumed thrust faulting for all the historical earthquakes as previous research indicated that the 1905 Kangra earthquake was a thrust event^13^ and the geodetic measurements as well as current seismicity suggest that this region is experiencing high compression. The historical earthquakes (Table 2) were reported with large uncertainty in their magnitudes^9^ and hence we took average magnitude for historical seismic strain analysis. Considering all the historical earthquakes (Table 2) since 844 AD, seismic strain rate is − 9.7 ns/year with average magnitude and − 19.4 ns/year if highest reported magnitude is considered. As the length of historical catalogue increases, seismic strain rate decreases due to large uncertainty in the reliability and completeness of the available record.

Considering that previous studies suggest 500–600 years recurrence interval for large earthquakes, we considered earthquakes since 1501 (this excludes only 2 events of 844 and 1123 AD) listed in Table 2 for seismic strain analysis. Major historical earthquakes documented with reported magnitudes (Table 2) during this period are 1501 M_w_ 6.5–7.0, 1555 M_w_ 7.6–8.2, 1669 M_w_ 6.5–7.0, 1678 M_w_ 6.5–6.8, 1683 M_w_ 6.5–6.8, 1736 M_w_ 6.5–7.0, 1779 M_w_ 6.5–7.5 and 1784 M_w_ 6.5–7.5^6,9,10^, 1828 M_w_ 6.5–7.5, 1863 M_w_ 6, 1878 M_w_ 6.76, 1885 M_w_ 6.4, and 1905 M_w_ 7.8–8.0. During this period, two major earthquakes with M_w_ ~ 8.0 occurred in 1555 and 1905. However, 1905 event is well documented compared to the 1555 event. If we consider M_w_ 8.0 for 1555 earthquake, seismic strain rate is − 21.7 ns/year which is low compared to geodetic strain rate indicating aseismic deformation and with M_w_ 8.2^9^, the seismic strain rate is − 32.0 ns/year which is comparable to the geodetic strain rate.^10^ assumed the existence of an afterslip that releases 34% of the seismic moment and corrected the estimates of the 1555 earthquake to M_w_ of 8.5 for every ~ 600 years, hence the calculated seismic strain rate for 1555 earthquake with M_w_ 8.5 is − 69.8 ns/year. As the uncertainty in the magnitude of the 1555 earthquake is high, we assumed average M_w_ of 8.0 for which seismic strain rate is − 21.7 ns/year towards N 40° (Table 3; Fig. 4).

Seismic strain rates provide a record of brittle deformation unlike the geodetic strain rates which give both seismic and aseismic strain accumulation. Seismic strain rates obtained from historical records are usually either overestimated or underestimated depending on the earthquake occurrence during the catalogue interval relative to the average recurrence interval of large earthquakes in the region. To estimate reliable strain rates, the average recurrence interval must be shorter than the historical record. For a region with individual fault, information on complete earthquake cycle is required whereas for a region with multiple faults, historical record must be long enough to capture all phases of the seismic cycle across multiple faults. Further, while converting strain rates into seismic moment rates, uncertainties associated in estimating the long-term seismic moment rate from historical record must be accounted for. This involves the uncertainty associated with the equations used to calculate the seismic moment, assumption of seismogenic layer thickness and the length of the catalogues.^41^ analyzed in detail, the factors that contribute to uncertainties in estimates of long-term seismic moment rate from historical catalogues.

## Conclusions

Our GPS observation network (Table 1) with two years of new continuous data till 2021 gave updated new velocity field with reduced uncertainties. Observed arc normal convergence rate of 5–14 mm/year and arc parallel extension rate of 7 mm/year confirm that the region is experiencing active oblique deformation pattern which can be attributed to several factors (i) post seismic deformation of Muzaffarabad 2005 earthquake in northwest region of the valley, (ii) presence of active unmapped subsurface structures such as deep-rooted faults and (iii) deformation across the regional faults^16^.

Geodetic strain rates obtained from GPS velocities of 58 sites (Figs. 2 and 4) suggest that the region is under high compression with high geodetic strain rate at the northern edge of higher Himalaya which is attributed to the slip along sub surface basal decollement MHT (Main Himalayan Thrust) along which Indian plate subducts below the Eurasian plate. This result is consistent with previous studies^15,16^ using both GPS and broadband data for the past 25 years in the various transects of 2500 km Himalayan arc. The rate of geodetic strain depends on the amount of plate convergence and the accumulated strain since the last large event.

The seismic strain deduced for instrumental period is − 28.1 ns/year is lower than the mean geodetic strain rate of − 32.5 ns/year (Table 3). The geodetic strain rate appears larger than the seismic strain rate suggesting that accumulated strain has not yet been released by the earthquakes. The 2005 Muzaffarabad earthquake is the major earthquake during the instrumental period and the previous studies indicate that accumulated strain is partially released during this event^10,18,20^. Hence a longer period catalogue is required for estimating the seismic potential of this region as the seismogenic crust is tectonically complex, with rheological and geometrical heterogeneity in the region, which could lead to considerable variations in the recurrence time and severity of future disastrous earthquakes.

The seismic strain rate (Table 3) deduced from Historical seismicity earthquake catalogue is − 21.7 ns/year which is less as compared to the geodetic strain rate of − 32.5 ± 7 ns/year. This suggest that relatively higher amount of accumulated strain energy is stored compared to released seismic energy by historical earthquakes, which would be released by next potential earthquake or the region is currently experiencing aseismic deformation.

In India, estimates are based on limited historical data as the earthquakes prior to 1800 AD are not comprehensively compiled^42,43^. Major earthquake during historical period with large uncertainty in its magnitude is the 1555 earthquake. Hence if we consider its magnitude as 8.2, the seismic strain rate is − 32.0 ns/year which is comparable with geodetic strain rates. This implies that we need a reliable and complete historical catalogue to infer the seismic potential of this region with certainty.

The average rate of geodetic strain accumulation of − 32.5 ns/year yields a seismic moment build-up rate of ~ 7.6E + 18 Nm/year for the seismogenic volume (~ 499.5 × 388.5 × 20 km^3^) of the region using the analytical expression (Eq. 5) by^44^. The released rate of seismic moment (Eq. 13) considering M_w_ 8.2 for the 1555 Kashmir earthquake is 7.6E+18 Nm/year which is comparable with the seismic moment build-up rate. This indicates a recurrence interval of ~ 520 years which is consistent with previous studies^20,40^ to generate an earthquake of M_w_ 8.2.

In the absence of paleo-seismic data for surface rupture, if we assume that rupture longer than 150 km is unlikely and hence the magnitude of 1555 earthquake is taken to be M_w_ 8 yielding a seismic moment rate (Eq. 13) of $5.2E+18$ Nm/year which is lesser than the geodetic moment build-up rate (Eq. 5) of 7.6E + 18 Nm/year. Hence, the total accumulated strain budget is ~ 1E + 21 Nm in the past ~ 520 years suggesting that this region has the potential to generate at least one great earthquake of M_w_ > 8 in the near future.

## Methodology

### GPS data and analysis

Continuous GPS (cGPS) data of the study region covering the rupture zones of the 2005 Muzaffarabad earthquake and the 1905 Kangra earthquake (Table 1; Fig. 1) are used to derive the decadal ITRF 2014 velocities. We used several cGPS sites located in the rest of Himalaya and plate interior along with the IGS sites for the robust data analysis with better constraints. To begin with, quality check of the cGPS data is performed using TEQC software^45^ and then analysed using GAMIT/GLOBK software^46^ to obtain the loosely constrained daily solutions after minimizing errors associated due to satellite and receiver clock, atmosphere, phase center, multipath, cycle slips and phase ambiguities. cGPS data above 20 h daily duration with elevation cut off angle of 15º and sampling interval of 30 s is used for the analysis. These daily solutions are combined using GLORG to estimate the velocities with their associated uncertainties of cGPS sites in ITRF 2014 reference frame by stabilizing the positions and velocities of IGS sites to their pre-determined precise values. ITRF 2014 velocities are converted to India fixed velocities (Table 1; Fig. 1) using the Euler pole of rotation given by^47^. In addition to these GPS velocities, we used published GPS velocities of about 44 GPS sites (Table S1; Fig. 1) in this region to determine the geodetic crustal strain rates.

### Geodetic strain rate estimation

Surface GPS velocities and the associated uncertainties of about 58 GPS sites (Table 1; Table S1) are inverted to compute the two-dimensional gridded strain field^48^ using the Modified Least Square (MLS) approach^49,50^ with a grid-strain program^48^. Grid-strain allows user to introduce the scale factor to account for the distance of the GPS sites i.e., Experimental points (Eps) from grid nodes. The input to the grid strain software is GPS velocities and positions and the output is a strain-rate tensor. GPS velocities of our cGPS sites along with published velocities with less than 3 mm uncertainty in Ladakh, Kashmir^30,31^, Himachal^29^, Indus-Kohistan Suture zone^10^ and adjoining regions were used in the strain rate calculations. The published velocities were transformed to International Terrestrial Reference Frame ITRF14^51^ and then to the India Fixed reference frame using Euler pole parameters estimated by^47^.

The geodetic strain rates at each grid node along *x* and *y* axes are estimated from GPS velocities spatially distributed in the study region. For our study we choose 25 × 25 km grid with optimum scale factor of 80 km based on the trade-off (Fig. S1) curve between the scale factor and average uncertainty. Grid size is chosen based on the spatial resolution and distribution of experimental points (i.e. GPS sites) and the previous studies^16,48,52^ suggest the scale factor to be approximately 3 times the grid size. In this approach, displacement gradient components with errors are defined as

1 $$u=AI+e$$

where, *u* is the pseudo-observable vector, *A* is information about positions of Eps and *I* is the parameter vector containing components of the displacement L_ij_ and *e* is the residual vector.

Displacement gradient *L* is defined as

2 $$L=E+\Omega$$

where, E is strain rate tensor (*E* = Ɛ_ij_ = (∂_i_u_j_ + ∂_j_u_i_)/2), *Ω* (= ω_ij_ = (∂_i_u_j_-∂_j_u_i_)/2) is rotational part of *L*, *u*_*i*_ is the displacement. Since *E* is asymmetric, so there exists a matrix *V* that is diagonalized as

E_d_ = *V*^−1^*EV* in which E_d_ is a diagonal matrix. Diagonal matrix E_d_ gives Eigen values Ɛ_max_ and Ɛ_min_ i.e. maximum and minimum principal strains and corresponding Eigen vectors are principal strain directions. If principal strain rate is positive it corresponds to the extension, whereas negative strain rate represents compression in the region.

The data with large uncertainty have a small effect on estimates, hence the contribution of one or more Eps could be reduced or excluded with a weighting factor to reduce corresponding errors. Weight factor W is defined as

3 $$W=f\left(dn/do\right)=exp\left(-dn/do\right)$$

where d_n_ is the distance of reference point (Eps) and grid point, d_o_ is the smoothing parameter.

Following^48^ if d_n_ = d_o_ then

4 $$W1=0.37W$$

If an experiment point falls within scale factor (i.e., d_n_ < d_o_), the contribution is large (> 37%) and for d_n_ > d_o_ contribution is low (< 37%) for the strain rate.

The grid plane is subdivided into three equal 120° apertures centred on computation points. If at least one Eps is at a distance less than or equal to the scale factor d_o_ for each sector, the strain obtained is considered to be of high significance. If two of the three 120° regions contains an Eps at a distance less than or equal to the scale factor and if the spatial distribution of the Eps around the grid point is good, then the strain obtained is considered to be of mean significance. The estimated value of strain for the remaining cases is of no significance and hence not used.

We estimate the scalar geodetic moment rate from the maximum and minimum horizontal principal strain rates i.e.,${\varepsilon }_{1}{\prime}$, ${\varepsilon }_{2}{\prime}$ in the region using following analytical relation given by^44^

5 $${M}_{0}{\prime}=2\mu AHMax\left(\left|{\varepsilon }_{1}{\prime}\right|,\left|{\varepsilon }_{2}{\prime}\right|,\left|{\varepsilon }_{1}{\prime}+{\varepsilon }_{2}{\prime}\right|\right)$$

where $\mu$ is the rigidity modulus (3E + 10 N/m^2^) and AH is the volume of seismogenic zone.

### Seismic strain rate estimation

A comprehensive and complete catalogue of seismic events is required to draw the seismicity map of the study region. Complete catalogues are frequently unavailable due to several factors, including seismic station distribution and sensitivity of the instruments. We made significant effort to acquire detailed information of historical seismic activity as well as a complete, homogeneous record of recent earthquake activities in the study region during 1964–2021. To calculate the seismic strain rate, we used earthquake catalogue of 50 + years of instrumentational period as well as the available historical record since 844 AD. To find magnitude completeness and seismogenic thickness of crust using ZMAP^35^, we have used the existing earthquake data for instrumental period (1964–2021) from the revised International Seismological Centre (ISC, www.isc.ac.uk/iscbulletin/search/catalogue, last access: March 2022)^14^ catalogue and fault plane solutions from GCMT catalogue to derive the seismic moment tensors.

According to^38^, the strain rate tensor for earthquakes N that occurred in a volume *V* are calculated using

6 $${\varepsilon }_{ij}=\frac{1}{2\mu tV}{\sum }_{n=1}^{k}{M}_{ij}^{k}=\frac{1}{2\mu AHt}{\sum }_{n=1}^{k}{M}_{ij}^{k}$$

where ${\varepsilon }_{ij}$ is the ijth component of strain rate tensor, µ is the rigidity modulus (3E+10 N/m^2^), t is the time period of observation and ${M}_{ij}^{k}$ is the ijth component of the seismic moment tensor of kth earthquake. In the cartesian coordinate system (e:east, n:north, u:vertical), the seismic moment tensor ${M}_{ij}^{k}$^36^ is

7 $${M}_{nn}=-{M}_{o}\left(sin\delta cos\lambda sin2\Phi +sin2\delta sin\lambda {sin}^{2}\Phi \right)$$

8 $${M}_{ne}=+{M}_{o}\left(sin\delta cos\lambda cos2\Phi +\frac{1}{2}sin2\delta sin\lambda sin2\Phi \right)={M}_{en}$$

9 $${M}_{nu}=-{M}_{o}\left(cos\delta cos\lambda cos\Phi +cos2\delta sin\lambda sin\Phi \right)={M}_{un}$$

10 $${M}_{ee}=+{M}_{o}\left(sin\delta cos\lambda sin2\Phi -sin2\delta sin\lambda {cos}^{2}\Phi \right),$$

11 $${M}_{nn}=-{M}_{o}\left(cos\delta cos\lambda sin\Phi -cos2\delta sin\lambda cos\Phi \right)={M}_{ue}$$

12 $${M}_{uu}=+{M}_{o}sin2\delta sin\lambda$$

where δ is dip, λ is rake and Φ is strike, are the fault parameters of the earthquake, M_o_ is seismic moment energy which is used to measure the deformation generated by earthquakes as given by^37^

13 $${M}_{w}=\frac{2}{3}{log}_{10}{M}_{o}-10.73$$

where M_w_ is moment magnitude of the earthquake. The eigen values and eigen vectors of the derived seismic strain tensor are used to calculate principal seismic strain rates and their orientations.

### Supplementary Information﻿

Supplementary Information.

## Data Availability

The datasets used and analysed during the current study are available from the corresponding author on reasonable request.
