# Supplementary material for: Implications of seismic and GNSS strain rates in Himachal, Kashmir and Ladakh
Source: Sci Rep. 2023 Dec 8;13:21652. doi: 10.1038/s41598-023-48997-3 (PMC10709641; doi:10.1038/s41598-023-48997-3)
Supplement: Supplementary file 1 — Supplementary Information. [file 41598_2023_48997_MOESM1_ESM.docx]

**Implications of Seismic and GNSS strain rates in Himachal, Kashmir and Ladakh**

**TS Shrungeshwara^1^, Bhavani Narukula^1^, Sridevi Jade^1 *^, Sapna Ghavri^2^, Chiranjeevi G Vivek ^1^ and I. A. Parvez^1^**

1 CSIR-4PI, CSIR Fourth Paradigm Institute (Formerly CSIR-CMMACS), Wind Tunnel Road, Bangalore 560037, India

2 Indian National Centre for Ocean Information Services (INCOIS), Ocean valley, JNTU Road, Nizampet, Hyderabad 500090, India

* Corresponding author: Tel: (+91) 80-25051929 Fax: (+91) 80-25220392

E-mail addresses:

[shrungeshsringeri@gmail.com](mailto:shrungeshsringeri@gmail.com) (TS Shrungeshwara); [bhavani.2728.narukula@gmail.com](mailto:bhavani.2728.narukula@gmail.com) (Bhavani Narukula); [sridevi@csir4pi.in](mailto:sridevi@csir4pi.in) (Sridevi Jade); [sapna.ngri@gmail.com](mailto:sapna.ngri@gmail.com) (Sapna Ghavri); [vivek@csir4pi.in](mailto:vivek@csir4pi.in) (Chiranjeevi G Vivek); [parvez@csir4pi.in](mailto:parvez@csir4pi.in) (I.A.Parvez)

**Supplementary Information**

Supplementary information contains

Figure S1: Trade-off curve between the scale factor and uncertainty in the strain rate.

Figure S2: Arc normal and Arc parallel velocities determined by rotating the site velocities to the local arc geometry for all the GPS sites used for strain computation.

Figure S3: Geodetic strain rates and dilatation of study region after removing 3 GPS points (0705, 0707, PM01) in Salt Range.

Table S1: Published GPS velocities included for Geodetic Strain Computation. Velocities are converted to International Terrestrial Reference Frame 2014 (ITRF14) and then to India fixed reference frame.

Table S2: Focal mechanism of earthquakes (M_w_≥3.9) in the study region.

Table S3: Calculated principal strain rates at the high and mean significance grid points.


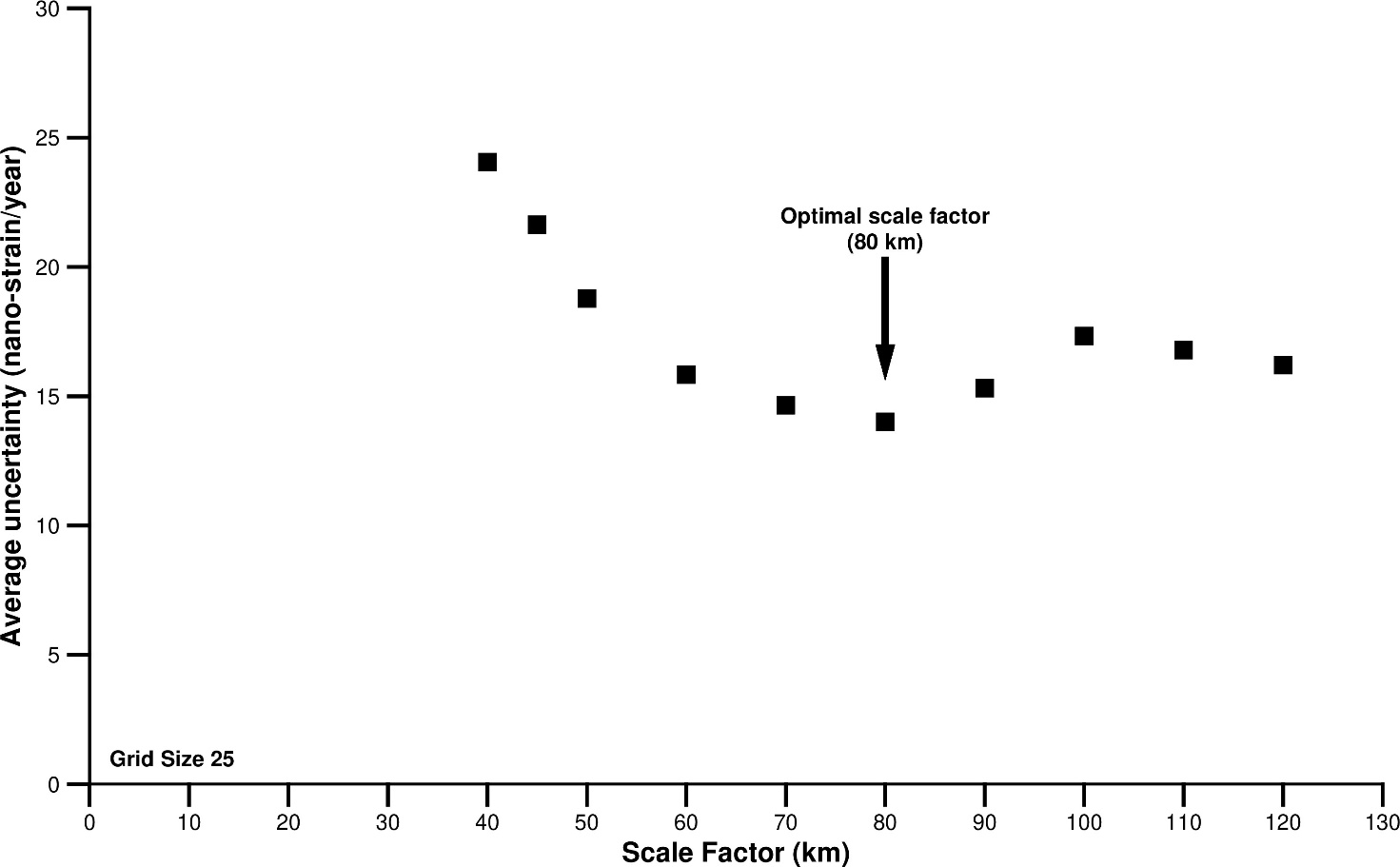


Figure S1: Trade-off curve between the scale factor and average uncertainty in the principal strain rate for a grid size of 25 km x 25 km. An optimal value of scale factor of 80 km is used to calculate the geodetic strain rate in the region. Figure was created using GMT (Generic Mapping Tool) software version 6.0.0[1].


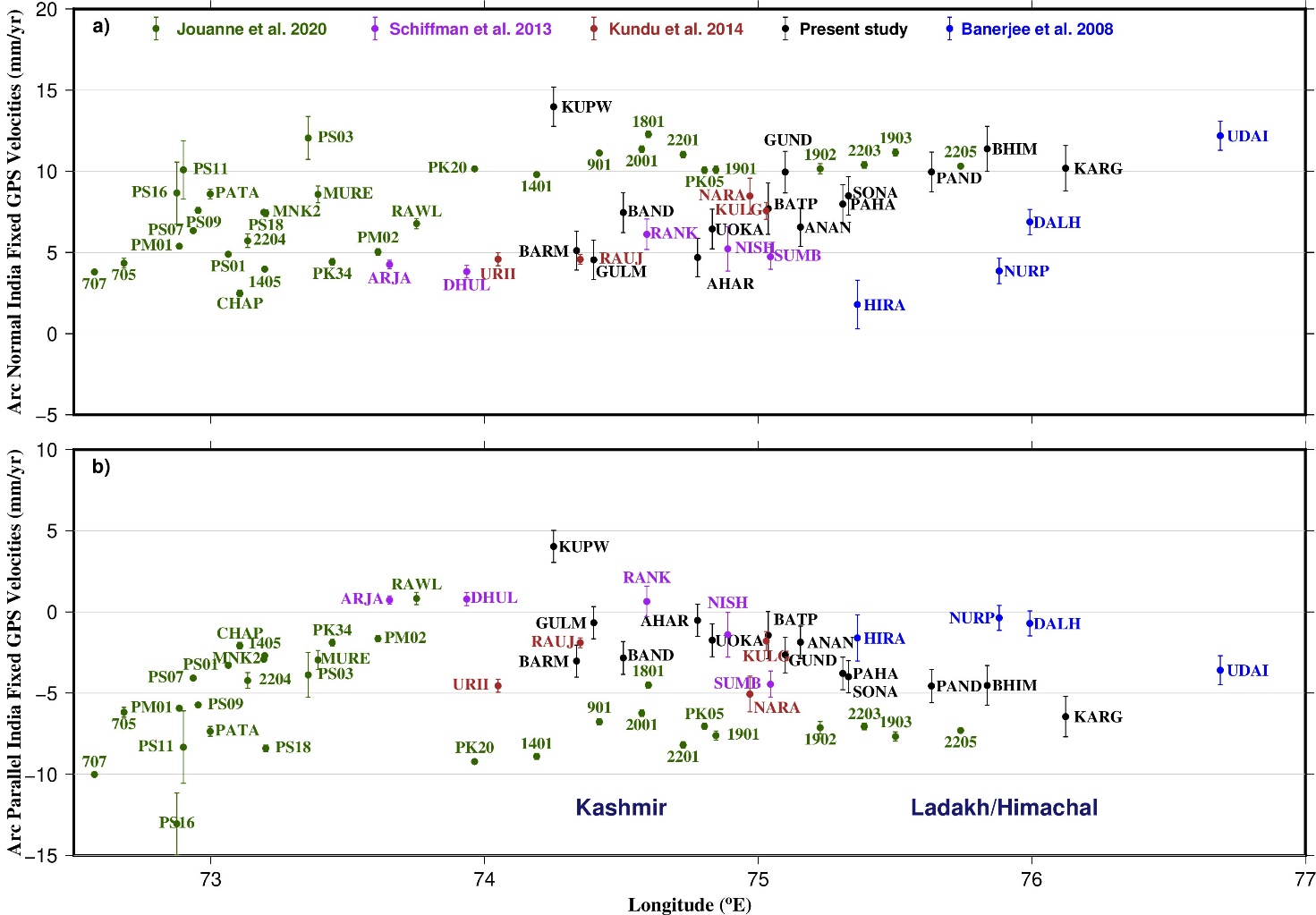


Figure S2: a) Arc-normal rates of GPS sites in Northwest Himalaya with error bars determined by rotating the site velocities on to the direction locally orthogonal to the arc using arc geometry defined by [2]. Figure was created using GMT (Generic Mapping Tool) software version 6.0.0[1].

b) Arc-parallel rates of GPS sites in Northwest Himalaya with error bars determined by rotating the site velocities onto the direction locally parallel to the arc using arc geometry defined by [2]. Figure was created using GMT (Generic Mapping Tool) software version 6.0.0[1].


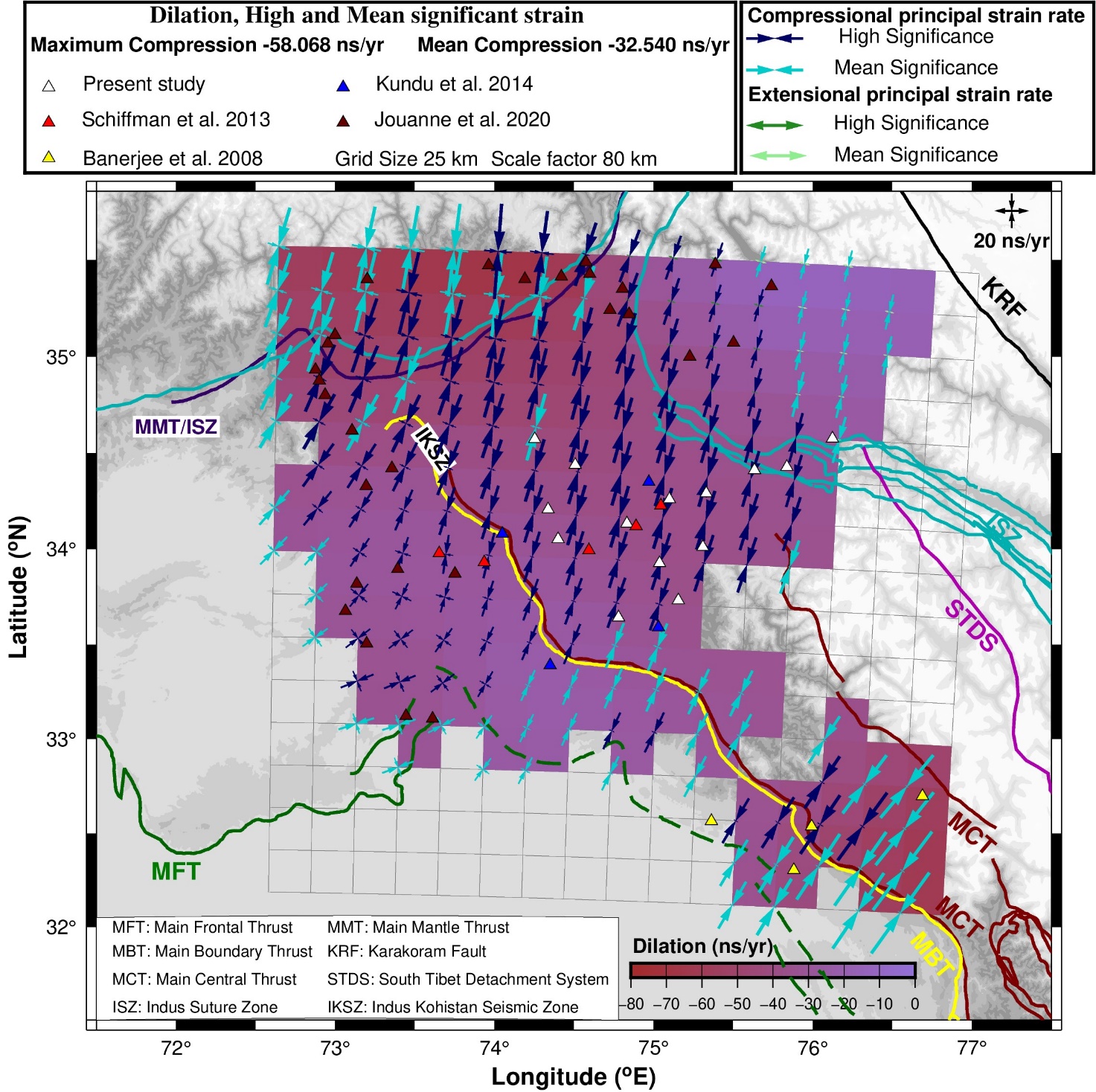


Figure S3: Geodetic strain rates and dilatation of study region after removing 3 GPS points (0705, 0707, PM01) in Salt Range. GPS site locations are denoted by solid triangles. The Figure was created using GMT (Generic Mapping Tool) software version 6.0.0[1]

Table S1: ITRF & India Fixed rates of GPS sites used in the analysis from published studies.

| **Site Code** | **Lat (^o^N)** | **Lon (^o^E)** | **ITRF14 velocities (mm/yr)** | | | | **India Fixed Velocities (mm/yr)** | | | |
| --- | --- | --- | --- | --- | --- | --- | --- | --- | --- | --- |
|  |  |  | **N** | **ᓂN** | **E** | **ᓂE** | **N** | **ᓂN** | **E** | **ᓂE** |
| HIRA^^^ | 32.57 | 75.36 | 32.31 | 1.37 | 33.25 | 1.54 | -2.31 | 1.37 | -0.67 | 1.54 |
| DALH^^^ | 32.54 | 75.99 | 30.30 | 0.76 | 28.79 | 0.78 | -4.39 | 0.76 | -5.35 | 0.78 |
| UDAI^^^ | 32.70 | 76.69 | 24.88 | 0.89 | 26.28 | 0.91 | -9.88 | 0.89 | -8.00 | 0.91 |
| NURP^^^ | 32.30 | 75.88 | 32.22 | 0.77 | 31.22 | 0.78 | -2.46 | 0.77 | -3.00 | 0.78 |
|  | | | | | | | | | | |
| ARJA^x^ | 33.98 | 73.65 | 33.27 | 0.26 | 28.45 | 0.26 | -1.14 | 0.26 | -4.17 | 0.26 |
| DHUL^x^ | 33.93 | 73.94 | 33.51 | 0.41 | 28.95 | 0.38 | -0.94 | 0.41 | -3.79 | 0.38 |
| RANK^x^ | 33.99 | 74.59 | 32.36 | 0.94 | 27.16 | 0.94 | -2.17 | 0.94 | -5.76 | 0.94 |
| NISH^x^ | 34.12 | 74.89 | 30.96 | 1.39 | 28.93 | 1.36 | -3.61 | 1.39 | -4.03 | 1.36 |
| SUMB^x^ | 34.23 | 75.05 | 28.47 | 0.82 | 30.73 | 0.76 | -6.11 | 0.82 | -2.22 | 0.76 |
|  | | | | | | | | | | |
| KULG^#^ | 33.59 | 75.03 | 29.38 | 0.60 | 27.50 | 0.50 | -5.20 | 0.60 | -5.78 | 0.50 |
| NARA^#^ | 34.35 | 74.97 | 26.28 | 1.10 | 27.50 | 1.10 | -8.29 | 1.10 | -5.36 | 1.10 |
| RAUJ^#^ | 33.39 | 74.35 | 30.68 | 0.30 | 30.00 | 0.30 | -3.82 | 0.30 | -3.16 | 0.30 |
| URII^#^ | 34.08 | 74.05 | 28.38 | 0.40 | 30.50 | 0.40 | -6.08 | 0.40 | -2.19 | 0.40 |
|  | | | | | | | | | | |
| 0705^*^ | 32.73 | 72.68 | 26.81 | 0.30 | 31.87 | 0.30 | -7.47 | 0.30 | -1.10 | 0.30 |
| 0707^*^ | 32.65 | 72.58 | 23.62 | 0.10 | 34.09 | 0.10 | -10.65 | 0.10 | 1.11 | 0.10 |
| 0901^*^ | 35.41 | 74.42 | 24.07 | 0.20 | 24.31 | 0.10 | -10.44 | 0.20 | -7.79 | 0.10 |
| 1401^*^ | 35.40 | 74.19 | 22.60 | 0.20 | 26.20 | 0.10 | -11.88 | 0.20 | -5.83 | 0.10 |
| 1405^*^ | 33.50 | 73.20 | 30.20 | 0.10 | 30.29 | 0.10 | -4.15 | 0.10 | -2.43 | 0.10 |
| 1801^*^ | 35.43 | 74.60 | 25.71 | 0.20 | 22.49 | 0.20 | -8.82 | 0.20 | -9.66 | 0.20 |
| 1901^*^ | 35.22 | 74.85 | 23.57 | 0.30 | 26.10 | 0.20 | -10.99 | 0.30 | -6.25 | 0.20 |
| 1902^*^ | 35.00 | 75.23 | 23.87 | 0.40 | 26.36 | 0.30 | -10.73 | 0.40 | -6.24 | 0.30 |
| 1903^*^ | 35.07 | 75.50 | 22.96 | 0.30 | 25.79 | 0.20 | -11.67 | 0.30 | -6.87 | 0.20 |
| 2001^*^ | 35.49 | 74.58 | 24.49 | 0.20 | 23.91 | 0.20 | -10.04 | 0.20 | -8.20 | 0.20 |
| 2201^*^ | 35.24 | 74.73 | 22.68 | 0.20 | 25.35 | 0.20 | -11.86 | 0.20 | -6.95 | 0.20 |
| 2203^*^ | 35.48 | 75.39 | 24.01 | 0.20 | 25.67 | 0.20 | -10.61 | 0.20 | -6.73 | 0.20 |
| 2204^*^ | 33.82 | 73.14 | 28.12 | 0.50 | 29.08 | 0.40 | -6.22 | 0.50 | -3.45 | 0.40 |
| 2205^*^ | 35.36 | 75.74 | 23.74 | 0.10 | 26.22 | 0.10 | -10.92 | 0.10 | -6.36 | 0.10 |
| CHAP^*^ | 34.62 | 73.11 | 31.48 | 0.20 | 30.57 | 0.20 | -2.86 | 0.20 | -1.51 | 0.20 |
| MNK2^*^ | 34.33 | 73.19 | 28.72 | 0.10 | 26.55 | 0.10 | -5.63 | 0.10 | -5.72 | 0.10 |
| MURE^*^ | 33.90 | 73.39 | 28.10 | 0.60 | 26.02 | 0.50 | -6.28 | 0.60 | -6.55 | 0.50 |
| PATA^*^ | 35.11 | 73.00 | 24.41 | 0.30 | 26.30 | 0.30 | -9.91 | 0.30 | -5.47 | 0.30 |
| PK05^*^ | 35.35 | 74.80 | 24.15 | 0.20 | 25.72 | 0.20 | -10.40 | 0.20 | -6.55 | 0.20 |
| PK20^*^ | 35.47 | 73.96 | 22.20 | 0.10 | 25.74 | 0.10 | -12.25 | 0.10 | -6.17 | 0.10 |
| PK34^*^ | 33.12 | 73.44 | 30.69 | 0.20 | 29.93 | 0.20 | -3.70 | 0.20 | -3.08 | 0.20 |
| PM01^*^ | 32.74 | 72.89 | 26.58 | 0.10 | 30.93 | 0.10 | -7.73 | 0.10 | -2.10 | 0.10 |
| PM02^*^ | 33.11 | 73.61 | 30.63 | 0.20 | 29.35 | 0.20 | -3.78 | 0.20 | -3.72 | 0.20 |
| PS01^*^ | 33.68 | 73.06 | 29.29 | 0.10 | 29.52 | 0.10 | -5.04 | 0.10 | -3.06 | 0.10 |
| PS03^*^ | 34.42 | 73.36 | 26.05 | 1.40 | 22.73 | 1.30 | -8.32 | 1.40 | -9.55 | 1.30 |
| PS07^*^ | 34.80 | 72.94 | 28.20 | 0.10 | 27.50 | 0.10 | -6.11 | 0.10 | -4.42 | 0.10 |
| PS09^*^ | 35.07 | 72.95 | 26.27 | 0.10 | 26.70 | 0.20 | -8.04 | 0.10 | -5.08 | 0.20 |
| PS11^*^ | 34.88 | 72.90 | 22.90 | 2.30 | 25.46 | 1.70 | -11.41 | 2.30 | -6.41 | 1.70 |
| PS16^*^ | 34.93 | 72.88 | 19.02 | 1.90 | 28.40 | 1.90 | -15.28 | 1.90 | -3.43 | 1.90 |
| PS18^*^ | 35.40 | 73.20 | 23.92 | 0.20 | 27.58 | 0.20 | -10.43 | 0.20 | -4.10 | 0.20 |
| RAWL^*^ | 33.87 | 73.75 | 32.25 | 0.40 | 26.23 | 0.30 | -2.17 | 0.40 | -6.48 | 0.30 |

^ [3]; x [4]; # [5] and * [6]

Table S2: Focal mechanisms of earthquakes (M_w_≥3.9) in the study region.

| **Lon**  **(^o^E)** | **Lat**  **(^o^N)** | **yyyy-mm-dd** | **Depth**  **(km)** | **M_w_** | **Strike 1** | **Dip1** | **Rake 1** | **Strike 2** | **Dip 2** | **Rake 2** | **Reference** |
| --- | --- | --- | --- | --- | --- | --- | --- | --- | --- | --- | --- |
| 75.37 | 32.59 | 1980-08-23 | 15 | 5.5 | 293 | 10 | 63 | 140 | 81 | 95 | GCMT |
| 75.40 | 32.49 | 1980-08-23 | 15 | 5.5 | 298 | 12 | 82 | 126 | 78 | 92 | GCMT |
| 73.48 | 35.22 | 1981-09-12 | 10 | 6.1 | 107 | 36 | 79 | 300 | 54 | 98 | GCMT |
| 73.40 | 35.15 | 1982-02-22 | 15 | 5.2 | 123 | 43 | 76 | 322 | 48 | 103 | GCMT |
| 74.45 | 35.13 | 1992-01-24 | 103.4 | 5.4 | 268 | 86 | 0 | 358 | 90 | -176 | GCMT |
| 73.14 | 32.61 | 2001-07-16 | 85.2 | 5.1 | 65 | 36 | 49 | 292 | 64 | 116 | GCMT |
| 75.46 | 33.00 | 2001-09-28 | 40.5 | 4.9 | 323 | 32 | 105 | 125 | 59 | 81 | GCMT |
| 75.91 | 33.32 | 2002-01-27 | 28.8 | 5.2 | 225 | 20 | -11 | 325 | 86 | -110 | GCMT |
| 74.64 | 35.36 | 2002-11-01 | 33.0 | 5.3 | 216 | 42 | -90 | 36 | 48 | -90 | GCMT |
| 74.70 | 35.10 | 2002-11-03 | 16.8 | 5.3 | 45 | 30 | -69 | 201 | 62 | -102 | GCMT |
| 73.22 | 34.75 | 2004-02-14 | 12 | 5.4 | 111 | 49 | 57 | 336 | 51 | 122 | GCMT |
| 73.12 | 34.78 | 2004-02-14 | 19 | 5.3 | 339 | 40 | 119 | 123 | 56 | 68 | GCMT |
| 74.44 | 34.91 | 2004-10-31 | 20.3 | 4.7 | 247 | 22 | -92 | 70 | 68 | -89 | GCMT |
| 74.37 | 35.20 | 2004-10-31 | 14.7 | 4.9 | 39 | 74 | -164 | 304 | 75 | -16 | GCMT |
| 74.34 | 35.16 | 2004-10-31 | 12 | 5.1 | 253 | 36 | -65 | 42 | 58 | -107 | GCMT |
| 73.47 | 34.38 | 2005-10-08 | 12 | 7.6 | 334 | 40 | 123 | 114 | 57 | 65 | GCMT |
| 73.12 | 34.70 | 2005-10-08 | 12 | 6.4 | 328 | 39 | 107 | 127 | 53 | 77 | GCMT |
| 73.06 | 34.58 | 2005-10-08 | 14.3 | 5.6 | 321 | 31 | 108 | 121 | 60 | 80 | GCMT |
| 73.10 | 34.68 | 2005-10-08 | 12 | 5.5 | 8 | 41 | 118 | 153 | 54 | 68 | GCMT |
| 73.06 | 34.60 | 2005-10-08 | 12 | 5.4 | 338 | 33 | 121 | 122 | 62 | 71 | GCMT |
| 72.86 | 34.69 | 2005-10-08 | 12 | 4.9 | 279 | 25 | 33 | 159 | 77 | 112 | GCMT |
| 73.17 | 34.93 | 2005-10-08 | 12 | 4.8 | 354 | 23 | 116 | 145 | 69 | 79 | GCMT |
| 73.34 | 34.62 | 2005-10-08 | 17.2 | 5.7 | 96 | 47 | 55 | 321 | 53 | 122 | GCMT |
| 72.94 | 34.70 | 2005-10-09 | 12 | 5.0 | 282 | 28 | 87 | 105 | 62 | 92 | GCMT |
| 73.01 | 34.57 | 2005-10-09 | 12 | 5.3 | 315 | 29 | 104 | 119 | 62 | 82 | GCMT |
| 73.13 | 34.65 | 2005-10-09 | 12 | 5.7 | 344 | 37 | 121 | 127 | 59 | 69 | GCMT |
| 72.86 | 34.64 | 2005-10-09 | 12 | 4.8 | 279 | 21 | 42 | 150 | 76 | 106 | GCMT |
| 73.26 | 34.60 | 2005-10-09 | 12 | 4.9 | 151 | 62 | 154 | 254 | 67 | 31 | GCMT |
| 73.02 | 34.55 | 2005-10-09 | 15.7 | 5.0 | 328 | 22 | 130 | 105 | 73 | 75 | GCMT |
| 73.03 | 34.74 | 2005-10-09 | 12 | 4.9 | 284 | 38 | 107 | 82 | 54 | 77 | GCMT |
| 73.58 | 34.33 | 2005-10-09 | 12 | 5.3 | 286 | 37 | 104 | 89 | 54 | 79 | GCMT |
| 72.87 | 34.62 | 2005-10-09 | 17 | 4.9 | 257 | 47 | 63 | 114 | 49 | 116 | GCMT |
| 73.26 | 34.65 | 2005-10-10 | 12 | 4.9 | 307 | 43 | 137 | 71 | 62 | 56 | GCMT |
| 73.08 | 34.88 | 2005-10-12 | 12 | 5.3 | 315 | 42 | 97 | 126 | 49 | 84 | GCMT |
| 73.10 | 34.69 | 2005-10-13 | 13.6 | 4.9 | 349 | 40 | 122 | 131 | 57 | 66 | GCMT |
| 73.00 | 34.91 | 2005-10-14 | 20.4 | 4.8 | 117 | 33 | 49 | 344 | 66 | 113 | GCMT |
| 73.08 | 34.81 | 2005-10-17 | 21.2 | 4.9 | 314 | 40 | 104 | 116 | 51 | 79 | GCMT |
| 73.09 | 34.72 | 2005-10-18 | 12 | 5.0 | 299 | 32 | 103 | 104 | 59 | 82 | GCMT |
| 73.05 | 34.76 | 2005-10-19 | 12 | 5.6 | 303 | 31 | 105 | 106 | 60 | 81 | GCMT |
| 73.14 | 34.78 | 2005-10-19 | 12 | 5.4 | 308 | 44 | 103 | 110 | 47 | 78 | GCMT |
| 72.73 | 34.98 | 2005-10-19 | 12 | 5.1 | 306 | 33 | 56 | 165 | 63 | 110 | GCMT |
| 73.03 | 34.74 | 2005-10-23 | 12 | 5.4 | 307 | 32 | 110 | 105 | 60 | 78 | GCMT |
| 73.11 | 34.75 | 2005-10-23 | 12 | 4.8 | 293 | 34 | 123 | 75 | 62 | 70 | GCMT |
| 73.18 | 34.76 | 2005-10-24 | 16.9 | 4.8 | 68 | 43 | 54 | 292 | 57 | 118 | GCMT |
| 73.85 | 34.11 | 2005-10-26 | 21.9 | 4.8 | 266 | 32 | 41 | 140 | 70 | 115 | GCMT |
| 73.15 | 34.64 | 2005-10-28 | 12 | 5.2 | 357 | 38 | 132 | 129 | 63 | 63 | GCMT |
| 73.20 | 34.51 | 2005-11-06 | 13 | 5.1 | 274 | 14 | 34 | 151 | 82 | 102 | GCMT |
| 73.12 | 34.68 | 2005-11-20 | 20.5 | 4.8 | 345 | 42 | 106 | 144 | 50 | 76 | GCMT |
| 73.19 | 34.67 | 2005-11-21 | 12 | 5.2 | 341 | 55 | 159 | 83 | 73 | 37 | GCMT |
| 73.14 | 34.79 | 2005-12-01 | 13.2 | 4.7 | 104 | 41 | 103 | 268 | 50 | 79 | GCMT |
| 73.20 | 34.65 | 2005-12-25 | 15.2 | 5.2 | 5 | 10 | 170 | 105 | 88 | 80 | GCMT |
| 73.15 | 34.84 | 2005-12-28 | 28.6 | 5.1 | 12 | 71 | -174 | 281 | 84 | -19 | GCMT |
| 73.18 | 34.83 | 2006-02-02 | 18.8 | 4.9 | 160 | 42 | 120 | 302 | 54 | 66 | GCMT |
| 72.99 | 34.75 | 2006-02-08 | 33.1 | 4.8 | 306 | 33 | 84 | 133 | 57 | 94 | GCMT |
| 73.51 | 32.62 | 2006-03-10 | 24.7 | 4.9 | 246 | 21 | 61 | 96 | 71 | 101 | GCMT |
| 73.03 | 34.69 | 2006-03-19 | 22.7 | 4.9 | 319 | 35 | 91 | 138 | 55 | 89 | GCMT |
| 73.57 | 34.75 | 2006-03-20 | 12 | 5.2 | 185 | 47 | 32 | 72 | 67 | 132 | GCMT |
| 73.22 | 34.52 | 2006-04-04 | 12 | 4.6 | 98 | 44 | 57 | 320 | 54 | 117 | GCMT |
| 76.62 | 35.27 | 2007-10-26 | 12 | 5.2 | 274 | 66 | -10 | 8 | 80 | -156 | GCMT |
| 73.79 | 34.14 | 2009-02-20 | 17.6 | 5.5 | 308 | 21 | 99 | 118 | 70 | 87 | GCMT |
| 73.01 | 34.93 | 2009-07-27 | 25.9 | 5.0 | 338 | 34 | 130 | 113 | 65 | 67 | GCMT |
| 73.75 | 34.88 | 2012-08-13 | 18.1 | 5.0 | 239 | 38 | 136 | 7 | 65 | 61 | GCMT |
| 75.60 | 33.02 | 2013-05-01 | 20.1 | 5.6 | 328 | 23 | 113 | 124 | 69 | 81 | GCMT |
| 75.95 | 33.09 | 2013-08-02 | 22.1 | 5.1 | 327 | 42 | 122 | 106 | 55 | 64 | GCMT |
| 75.71 | 33.10 | 2013-08-02 | 25.9 | 5.1 | 319 | 28 | 124 | 101 | 67 | 73 | GCMT |
| 76.95 | 35.44 | 2013-10-21 | 16.5 | 5.6 | 3 | 63 | -160 | 264 | 72 | -28 | GCMT |
| 73.28 | 34.57 | 2015-02-26 | 19.7 | 5.1 | 301 | 37 | 87 | 125 | 53 | 92 | GCMT |
| 73.21 | 33.92 | 2015-07-24 | 22.1 | 5.0 | 148 | 82 | 6 | 58 | 84 | 172 | GCMT |
| 73.54 | 34.82 | 2016-10-01 | 16.9 | 5.1 | 219 | 36 | 91 | 37 | 54 | 89 | GCMT |
| 74.60 | 35.24 | 2017-08-23 | 12 | 4.7 | 267 | 39 | -33 | 24 | 70 | -125 | GCMT |
| 74.20 | 35.11 | 2019-02-05 | 19.1 | 5.3 | 242 | 49 | 154 | 350 | 71 | 44 | GCMT |
| 75.87 | 32.92 | 2019-09-09 | 30.1 | 4.9 | 300 | 35 | 92 | 118 | 55 | 89 | GCMT |
| 73.85 | 32.83 | 2019-09-24 | 14.7 | 5.7 | 246 | 10 | 52 | 105 | 82 | 96 | GCMT |
| 74.52 | 35.49 | 2019-12-30 | 12 | 5.1 | 59 | 46 | -55 | 193 | 54 | -121 | GCMT |

Table S3: Strain rate at high and mean significance grid points. The maximum strain Emax is the change of length per unit-length in the direction of maximum extension (Positive for extension, Negative for compression)

| **SN** | **Lon (^o^E)** | **Lat (^o^N)** | **Principal Strain rate (x10^-9^)** | | | | **Azimuth of Emin (^o^N)** | **Dilation (x 10^-9^)** |
| --- | --- | --- | --- | --- | --- | --- | --- | --- |
|  |  |  | **Emax** | **Emin** | **eEmax** | **eEmin** |  |  |
| **Strain rate at high significance grid points** | | | | | | | | |
|  |  |  |  |  |  |  |  |  |
| HSG01 | 72.917 | 34.223 | -9.67 | -18.64 | 0.34 | 10.09 | 59.39 | -28.31 |
| HSG02 | 72.922 | 34.448 | -9.12 | -25.66 | 1.00 | 9.49 | 43.27 | -34.77 |
| HSG03 | 72.927 | 34.674 | -7.89 | -33.21 | 1.76 | 8.13 | 32.89 | -41.10 |
| HSG04 | 73.932 | 34.899 | -7.50 | -39.83 | 2.03 | 6.61 | 25.93 | -47.34 |
| HSG05 | 73.160 | 33.091 | 22.36 | -37.57 | 16.95 | 50.84 | 113.68 | -15.21 |
| HSG06 | 73.165 | 33.317 | 17.14 | -30.80 | 18.22 | 54.67 | 114.43 | -13.66 |
| HSG07 | 73.171 | 33.542 | 7.62 | -23.55 | 20.39 | 61.17 | 116.46 | -15.92 |
| HSG08 | 73.176 | 33.767 | -2.29 | -18.13 | 20.04 | 60.13 | 115.44 | -20.42 |
| HSG09 | 73.182 | 33.993 | -9.72 | -13.50 | 12.34 | 37.02 | 97.77 | -23.22 |
| HSG10 | 73.188 | 34.218 | -8.11 | -18.80 | 1.27 | 8.85 | 40.56 | -26.90 |
| HSG11 | 73.194 | 34.444 | -8.36 | -26.70 | 1.50 | 8.68 | 37.60 | -35.06 |
| HSG12 | 73.212 | 35.120 | -10.78 | -43.41 | 1.92 | 5.38 | 20.46 | -54.19 |
| HSG13 | 73.218 | 35.345 | -15.34 | -45.76 | 1.90 | 4.90 | 15.91 | -61.10 |
| HSG14 | 73.427 | 33.086 | 18.63 | -32.43 | 5.37 | 2.51 | 112.92 | -13.80 |
| HSG15 | 73.434 | 33.312 | 11.97 | -26.37 | 16.96 | 50.89 | 111.96 | -14.40 |
| HSG16 | 73.440 | 33.537 | 2.73 | -19.82 | 18.12 | 54.35 | 111.80 | -17.09 |
| HSG17 | 73.446 | 33.762 | -5.19 | -15.62 | 19.66 | 58.99 | 114.63 | -20.81 |
| HSG18 | 73.453 | 33.988 | -10.95 | -12.69 | 7.95 | 1.67 | 165.02 | -23.64 |
| HSG19 | 73.459 | 34.213 | -6.92 | -20.57 | 2.58 | 6.83 | 26.30 | -27.49 |
| HSG20 | 73.466 | 34.438 | -6.47 | -28.46 | 2.03 | 6.95 | 29.53 | -34.93 |
| HSG21 | 73.473 | 34.664 | -8.13 | -34.82 | 1.88 | 6.39 | 28.23 | -42.95 |
| HSG22 | 73.479 | 34.889 | -9.75 | -39.66 | 1.88 | 5.67 | 24.00 | -49.40 |
| HSG23 | 73.486 | 35.114 | -12.11 | -43.47 | 1.91 | 5.25 | 19.33 | -55.58 |
| HSG24 | 73.493 | 35.340 | -15.05 | -46.80 | 1.97 | 5.10 | 14.84 | -61.85 |
| HSG25 | 73.702 | 33.306 | 6.19 | -20.15 | 16.25 | 48.74 | 109.25 | -13.96 |
| HSG26 | 73.709 | 33.531 | -1.49 | -14.92 | 16.34 | 49.03 | 107.13 | -16.42 |
| HSG27 | 73.716 | 33.757 | -8.26 | -12.70 | 6.96 | 2.44 | 117.59 | -20.96 |
| HSG28 | 73.723 | 33.982 | -9.01 | -16.46 | 17.11 | 51.32 | 1.51 | -25.47 |
| HSG29 | 73.730 | 34.207 | -5.50 | -24.44 | 9.11 | 27.33 | 19.88 | -29.94 |
| HSG30 | 73.738 | 34.433 | -5.47 | -31.03 | 2.30 | 5.88 | 24.07 | -36.50 |
| HSG31 | 73.745 | 34.658 | -7.62 | -35.91 | 2.05 | 5.58 | 23.85 | -43.53 |
| HSG32 | 73.753 | 34.883 | -10.10 | -40.03 | 2.03 | 5.29 | 20.69 | -50.12 |
| HSG33 | 73.760 | 35.108 | -12.71 | -44.31 | 2.15 | 5.21 | 15.94 | -57.03 |
| HSG34 | 73.970 | 33.300 | 0.73 | -12.48 | 14.49 | 43.46 | 102.84 | -11.74 |
| HSG35 | 73.978 | 33.525 | -5.61 | -9.71 | 9.94 | 29.81 | 87.56 | -15.33 |
| HSG36 | 73.986 | 33.750 | -7.35 | -13.04 | 2.96 | 6.03 | 20.03 | -20.39 |
| HSG37 | 73.994 | 33.976 | -4.66 | -21.13 | 10.75 | 32.26 | 14.70 | -25.79 |
| HSG38 | 74.002 | 34.201 | -3.16 | -28.38 | 2.85 | 5.56 | 18.98 | -31.54 |
| HSG39 | 74.010 | 34.426 | -4.83 | -33.48 | 2.43 | 5.36 | 20.63 | -38.31 |
| HSG40 | 74.018 | 34.651 | -7.32 | -37.23 | 2.26 | 5.20 | 19.74 | -44.55 |
| HSG41 | 74.026 | 34.877 | -9.65 | -40.70 | 2.22 | 5.12 | 16.91 | -50.35 |
| HSG42 | 74.034 | 35.102 | -12.38 | -44.83 | 2.41 | 5.33 | 12.42 | -57.20 |
| HSG43 | 74.051 | 35.552 | -16.12 | -53.90 | 2.89 | 6.23 | 4.77 | -70.01 |
| HSG44 | 74.247 | 33.518 | -4.80 | -11.70 | 1.45 | 6.19 | 33.71 | -16.50 |
| HSG45 | 74.255 | 33.744 | -4.29 | -18.10 | 2.33 | 5.78 | 22.46 | -22.40 |
| HSG46 | 74.264 | 33.969 | -3.05 | -25.19 | 2.72 | 5.46 | 18.47 | -28.24 |
| HSG47 | 74.273 | 34.194 | -2.51 | -31.25 | 2.59 | 5.17 | 18.33 | -33.76 |
| HSG48 | 74.281 | 34.419 | -4.04 | -35.33 | 2.43 | 5.06 | 18.32 | -39.36 |
| HSG49 | 74.299 | 34.870 | -7.28 | -40.70 | 2.21 | 5.17 | 15.17 | -47.97 |
| HSG50 | 74.308 | 35.095 | -9.32 | -43.03 | 2.28 | 5.91 | 12.85 | -52.35 |
| HSG51 | 74.327 | 35.545 | -13.30 | -46.87 | 2.54 | 8.18 | 9.02 | -60.17 |
| HSG52 | 74.516 | 33.511 | -4.34 | -16.53 | 1.65 | 5.44 | 26.33 | -20.87 |
| HSG53 | 74.525 | 33.736 | -3.90 | -22.40 | 2.05 | 5.19 | 21.50 | -26.29 |
| HSG54 | 74.534 | 33.961 | -3.27 | -28.33 | 2.33 | 4.92 | 18.14 | -31.60 |
| HSG55 | 74.544 | 34.187 | -2.36 | -33.27 | 2.29 | 4.69 | 17.48 | -35.63 |
| HSG56 | 74.553 | 34.412 | -2.44 | -36.45 | 2.17 | 4.61 | 17.14 | -38.89 |
| HSG57 | 74.563 | 34.637 | -2.93 | -38.42 | 2.04 | 4.76 | 16.31 | -41.35 |
| HSG58 | 74.572 | 34.862 | -3.17 | -39.52 | 1.91 | 5.28 | 15.66 | -42.69 |
| HSG59 | 74.582 | 35.087 | -3.14 | -39.32 | 1.80 | 6.61 | 16.24 | -42.46 |
| HSG60 | 74.592 | 35.312 | -3.00 | -37.31 | 1.62 | 8.86 | 19.00 | -40.31 |
| HSG61 | 74.602 | 35.537 | -3.65 | -37.22 | 1.45 | 10.20 | 19.30 | -40.87 |
| HSG62 | 74.765 | 33.053 | -4.83 | -11.07 | 1.15 | 5.87 | 50.68 | -15.91 |
| HSG63 | 74.794 | 33.728 | -4.74 | -25.77 | 1.83 | 4.66 | 20.27 | -30.51 |
| HSG64 | 74.804 | 33.953 | -3.22 | -30.51 | 2.01 | 4.44 | 17.98 | -33.73 |
| HSG65 | 74.814 | 34.178 | -1.66 | -34.47 | 2.00 | 4.25 | 16.89 | -36.12 |
| HSG66 | 74.825 | 34.403 | -0.80 | -36.60 | 1.86 | 4.24 | 16.90 | -37.40 |
| HSG67 | 74.835 | 34.628 | -0.31 | -37.59 | 1.71 | 4.50 | 16.41 | -37.90 |
| HSG68 | 74.845 | 34.854 | 0.50 | -37.39 | 1.55 | 5.23 | 16.36 | -36.89 |
| HSG69 | 74.856 | 35.079 | 1.89 | -35.34 | 1.37 | 6.63 | 17.72 | -33.45 |
| HSG70 | 74.867 | 35.304 | 3.71 | -31.89 | 1.12 | 8.48 | 20.62 | -28.18 |
| HSG71 | 74.877 | 35.529 | 3.86 | -31.41 | 0.92 | 9.65 | 20.96 | -27.55 |
| HSG72 | 75.032 | 33.045 | -5.38 | -14.97 | 1.02 | 5.37 | 37.81 | -20.35 |
| HSG73 | 75.064 | 33.72 | -5.50 | -27.91 | 1.66 | 4.20 | 19.84 | -33.41 |
| HSG74 | 75.074 | 33.945 | -3.23 | -31.67 | 1.79 | 4.03 | 17.96 | -34.90 |
| HSG75 | 75.085 | 34.170 | -0.91 | -34.60 | 1.78 | 3.98 | 17.19 | -35.51 |
| HSG76 | 75.096 | 34.395 | 0.15 | -35.91 | 1.64 | 3.96 | 16.86 | -35.76 |
| HSG77 | 75.107 | 34.620 | 1.26 | -35.97 | 1.47 | 4.28 | 16.43 | -34.71 |
| HSG78 | 75.118 | 34.845 | 2.57 | -34.31 | 1.27 | 5.04 | 16.65 | -31.75 |
| HSG79 | 75.130 | 35.070 | 4.01 | -30.63 | 1.06 | 6.14 | 17.69 | -26.62 |
| HSG80 | 75.141 | 35.294 | 5.44 | -27.23 | 0.84 | 7.40 | 18.75 | -21.80 |
| HSG81 | 75.152 | 35.519 | 6.23 | -25.85 | 0.65 | 8.45 | 19.37 | -19.62 |
| HSG82 | 75.344 | 33.935 | -2.83 | -31.98 | 1.64 | 3.73 | 18.36 | -34.81 |
| HSG83 | 75.356 | 34.160 | -1.05 | -33.82 | 1.60 | 3.68 | 17.57 | -34.87 |
| HSG84 | 75.367 | 34.385 | 0.34 | -34.45 | 1.49 | 3.74 | 16.80 | -34.10 |
| HSG85 | 75.379 | 34.610 | 1.87 | -33.70 | 1.34 | 4.11 | 16.18 | -31.84 |
| HSG86 | 75.391 | 34.835 | 3.27 | -30.73 | 1.15 | 4.84 | 16.33 | -27.47 |
| HSG87 | 75.403 | 35.060 | 4.37 | -24.78 | 0.93 | 5.74 | 17.28 | -20.40 |
| HSG88 | 75.415 | 35.285 | 5.81 | -21.57 | 0.74 | 6.54 | 17.30 | -15.76 |
| HSG89 | 75.428 | 35.510 | 6.82 | -20.18 | 0.54 | 7.35 | 18.51 | -13.36 |
| HSG90 | 75.544 | 32.576 | -4.73 | -17.49 | 0.84 | 4.73 | 42.27 | -22.22 |
| HSG91 | 75.614 | 33.926 | -2.49 | -31.73 | 1.54 | 3.54 | 18.63 | -34.22 |
| HSG92 | 75.626 | 34.150 | -1.03 | -32.74 | 1.48 | 3.52 | 17.76 | -33.78 |
| HSG93 | 75.639 | 34.375 | 0.04 | -32.39 | 1.39 | 3.59 | 16.88 | -32.35 |
| HSG94 | 75.651 | 34.600 | 1.87 | -31.09 | 1.30 | 3.94 | 15.72 | -29.23 |
| HSG95 | 75.664 | 34.825 | 3.45 | -27.82 | 1.16 | 4.60 | 15.25 | -24.38 |
| HSG96 | 75.677 | 35.050 | 4.57 | -22.01 | 1.00 | 5.34 | 15.20 | -17.45 |
| HSG97 | 75.689 | 35.274 | 5.93 | -18.86 | 0.78 | 5.94 | 15.11 | -12.93 |
| HSG98 | 75.809 | 32.566 | -5.82 | -23.42 | 0.86 | 4.88 | 38.61 | -29.24 |
| HSG99 | 75.910 | 34.364 | 0.08 | -30.44 | 1.34 | 3.53 | 16.95 | -30.36 |
| HSG100 | 75.923 | 34.589 | 1.91 | -28.69 | 1.28 | 3.79 | 15.57 | -26.78 |
| HSG101 | 76.075 | 32.556 | -6.13 | -30.80 | 0.97 | 5.30 | 37.08 | -36.92 |
| HSG102 | 76.087 | 32.781 | -5.52 | -28.68 | 0.90 | 4.63 | 37.29 | -34.20 |
| HSG103 | 76.340 | 32.545 | -5.20 | -39.32 | 1.07 | 5.78 | 38.07 | -44.52 |
| **Strain rate at mean significance grid points** | | | | | | | | |
| MSG01 | 72.352 | 32.877 | 25.15 | -73.42 | 20.21 | 60.63 | 113.28 | -48.26 |
| MSG02 | 72.359 | 33.328 | 21.89 | -48.23 | 20.20 | 60.59 | 116.92 | -26.35 |
| MSG03 | 72.389 | 35.132 | -6.74 | -39.01 | 1.95 | 6.41 | 26.99 | -45.75 |
| MSG04 | 72.393 | 35.357 | -8.46 | -41.37 | 1.94 | 5.84 | 24.04 | -49.83 |
| MSG05 | 72.396 | 35.583 | -10.63 | -42.86 | 1.89 | 5.47 | 21.60 | -53.49 |
| MSG06 | 72.608 | 32.197 | 26.44 | -82.15 | 7.55 | 3.46 | 112.00 | -55.71 |
| MSG07 | 72.612 | 32.423 | 26.46 | -85.11 | 7.47 | 3.47 | 112.11 | -58.66 |
| MSG08 | 72.616 | 32.648 | 26.37 | -86.06 | 7.39 | 3.52 | 112.23 | -59.70 |
| MSG09 | 72.620 | 32.874 | 25.50 | -71.09 | 20.4 | 61.20 | 112.90 | -45.59 |
| MSG10 | 72.624 | 33.099 | 24.32 | -54.42 | 19.25 | 57.75 | 114.48 | -30.11 |
| MSG11 | 72.628 | 33.325 | 21.59 | -42.71 | 19.90 | 59.69 | 116.66 | -21.12 |
| MSG12 | 72.641 | 34.001 | -3.75 | -20.64 | 18.45 | 55.36 | 110.24 | -24.39 |
| MSG13 | 72.645 | 34.227 | -9.88 | -18.46 | 1.08 | 9.44 | 76.64 | -28.34 |
| MSG14 | 72.654 | 34.678 | -7.27 | -31.36 | 1.58 | 8.18 | 34.80 | -38.62 |
| MSG15 | 72.659 | 34.903 | -6.78 | -37.65 | 1.97 | 6.94 | 28.20 | -44.42 |
| MSG16 | 72.663 | 35.128 | -7.73 | -41.39 | 2.06 | 6.03 | 23.92 | -49.12 |
| MSG17 | 72.668 | 35.354 | -10.06 | -43.29 | 1.99 | 5.51 | 21.12 | -53.35 |
| MSG18 | 72.672 | 35.579 | -12.64 | -44.52 | 1.91 | 5.22 | 18.72 | -57.16 |
| MSG19 | 72.873 | 32.194 | 26.23 | -73.28 | 7.53 | 3.42 | 111.89 | -47.05 |
| MSG20 | 72.878 | 32.419 | 26.22 | -74.88 | 7.40 | 3.41 | 112.115 | -48.66 |
| MSG21 | 72.882 | 32.645 | 26.07 | -73.63 | 7.14 | 3.42 | 112.42 | -47.56 |
| MSG22 | 72.887 | 32.870 | 25.50 | -60.25 | 19.60 | 58.80 | 113.08 | -34.75 |
| MSG23 | 72.892 | 33.096 | 24.03 | -45.81 | 18.31 | 54.94 | 114.26 | -21.78 |
| MSG24 | 72.897 | 33.321 | 20.29 | -36.23 | 19.25 | 57.76 | 116.05 | -15.93 |
| MSG25 | 72.902 | 33.546 | 12.37 | -28.47 | 21.07 | 63.21 | 117.83 | -16.10 |
| MSG26 | 72.907 | 33.772 | 1.83 | -22.03 | 21.19 | 63.58 | 117.02 | -20.20 |
| MSG27 | 72.912 | 33.997 | -7.06 | -16.81 | 15.50 | 46.51 | 104.77 | -23.87 |
| MSG28 | 72.938 | 35.124 | -8.59 | -43.34 | 2.08 | 5.64 | 21.10 | -51.92 |
| MSG29 | 72.943 | 35.350 | -12.35 | -44.79 | 1.98 | 5.19 | 18.31 | -57.14 |
| MSG30 | 73.149 | 32.640 | 25.25 | -53.70 | 6.59 | 3.09 | 112.56 | -28.46 |
| MSG31 | 73.154 | 32.866 | 24.52 | -45.10 | 17.90 | 53.71 | 113.15 | -20.59 |
| MSG32 | 73.200 | 34.669 | -8.82 | -34.36 | 1.83 | 7.75 | 31.77 | -43.18 |
| MSG33 | 73.206 | 34.894 | -8.86 | -39.93 | 1.93 | 6.18 | 25.25 | -48.79 |
| MSG34 | 73.224 | 35.570 | -16.91 | -47.45 | 1.89 | 4.90 | 13.41 | -64.36 |
| MSG35 | 73.409 | 32.410 | 24.21 | -44.64 | 6.53 | 3.02 | 111.40 | -20.43 |
| MSG36 | 73.415 | 32.635 | 23.54 | -40.42 | 6.06 | 2.81 | 111.96 | -16.88 |
| MSG37 | 73.421 | 32.861 | 22.06 | -35.95 | 5.58 | 2.58 | 112.47 | -13.89 |
| MSG38 | 73.500 | 35.565 | -16.70 | -49.58 | 2.07 | 5.20 | 11.39 | -66.28 |
| MSG39 | 73.695 | 33.081 | 13.14 | -26.14 | 5.41 | 2.62 | 110.97 | -13.00 |
| MSG40 | 73.768 | 35.334 | -15.07 | -49.23 | 2.37 | 5.31 | 10.94 | -64.30 |
| MSG41 | 73.776 | 35.559 | -16.35 | -53.07 | 2.53 | 5.56 | 7.59 | -69.42 |
| MSG42 | 73.955 | 32.849 | 12.74 | -20.72 | 5.57 | 2.83 | 108.19 | -7.99 |
| MSG43 | 73.963 | 33.075 | 7.17 | -16.91 | 15.97 | 47.92 | 107.14 | -9.74 |
| MSG44 | 74.043 | 35.327 | -15.09 | -50.13 | 2.76 | 5.75 | 7.28 | -65.22 |
| MSG45 | 74.222 | 32.843 | 7.45 | -13.79 | 15.40 | 46.20 | 102.89 | -6.34 |
| MSG46 | 74.230 | 33.068 | 1.85 | -10.54 | 13.71 | 41.14 | 98.88 | -8.68 |
| MSG47 | 74.239 | 33.293 | -3.52 | -8.04 | 2.47 | 5.04 | 77.21 | -11.56 |
| MSG48 | 74.290 | 34.644 | -6.09 | -38.29 | 2.37 | 5.09 | 16.80 | -44.38 |
| MSG49 | 74.317 | 35.320 | -12.45 | -45.18 | 2.52 | 7.24 | 10.37 | -57.63 |
| MSG50 | 74.498 | 33.061 | -2.63 | -8.72 | 8.56 | 25.69 | 80.22 | -11.35 |
| MSG51 | 74.507 | 33.286 | -4.73 | -10.91 | 1.15 | 5.99 | 39.01 | -15.64 |
| MSG52 | 74.756 | 32.828 | -1.52 | -9.49 | 7.91 | 23.72 | 77.29 | -11.01 |
| MSG53 | 74.775 | 33.278 | -5.48 | -15.58 | 1.27 | 5.53 | 31.49 | -21.06 |
| MSG54 | 74.785 | 33.503 | -5.51 | -20.72 | 1.56 | 5.02 | 24.43 | -26.23 |
| MSG55 | 75.043 | 33.270 | -6.15 | -19.38 | 1.27 | 5.02 | 28.46 | -25.43 |
| MSG56 | 75.053 | 33.495 | -6.71 | -23.76 | 1.44 | 4.58 | 23.59 | -30.47 |
| MSG57 | 75.289 | 32.811 | -4.46 | -15.23 | 0.87 | 4.77 | 41.14 | -19.69 |
| MSG58 | 75.300 | 33.036 | -5.41 | -18.47 | 1.00 | 4.72 | 33.40 | -23.88 |
| MSG59 | 75.311 | 33.261 | -6.07 | -22.15 | 1.24 | 4.51 | 26.94 | -28.22 |
| MSG60 | 75.521 | 32.127 | -3.09 | -13.79 | 1.49 | 4.49 | 58.22 | -16.88 |
| MSG61 | 75.532 | 32.351 | -4.25 | -15.61 | 1.01 | 4.81 | 48.94 | -19.86 |
| MSG62 | 75.578 | 33.251 | -5.55 | -24.07 | 1.14 | 4.08 | 27.65 | -29.62 |
| MSG63 | 75.702 | 35.499 | 6.82 | -18.33 | 0.56 | 6.65 | 16.68 | -11.51 |
| MSG64 | 75.785 | 32.117 | -5.31 | -20.90 | 0.92 | 5.55 | 41.88 | -26.21 |
| MSG65 | 75.797 | 32.342 | -5.63 | -23.39 | 0.89 | 5.36 | 38.44 | -29.02 |
| MSG66 | 75.884 | 33.915 | -2.15 | -30.92 | 1.44 | 3.42 | 19.38 | -33.07 |
| MSG67 | 75.897 | 34.140 | -0.89 | -31.40 | 1.40 | 3.43 | 18.07 | -32.28 |
| MSG68 | 75.936 | 34.814 | 3.56 | -26.04 | 1.19 | 4.30 | 14.39 | -22.48 |
| MSG69 | 75.950 | 35.039 | 4.77 | -22.21 | 1.06 | 4.91 | 13.77 | -17.44 |
| MSG70 | 75.963 | 35.263 | 5.78 | -19.20 | 0.87 | 5.49 | 13.97 | -13.42 |
| MSG71 | 75.977 | 35.488 | 6.47 | -18.01 | 0.67 | 6.12 | 15.21 | -11.54 |
| MSG72 | 76.062 | 32.331 | -6.03 | -32.19 | 1.02 | 5.85 | 36.07 | -38.22 |
| MSG73 | 76.100 | 33.005 | -5.02 | -27.16 | 0.85 | 4.06 | 36.28 | -32.17 |
| MSG74 | 76.209 | 34.803 | 3.65 | -24.89 | 1.20 | 4.05 | 14.27 | -21.25 |
| MSG75 | 76.223 | 35.027 | 4.73 | -22.37 | 1.10 | 4.57 | 13.36 | -17.64 |
| MSG76 | 76.237 | 35.252 | 5.55 | -19.97 | 0.95 | 5.12 | 13.44 | -14.41 |
| MSG77 | 76.313 | 32.096 | -6.00 | -38.49 | 1.09 | 6.48 | 35.84 | -44.49 |
| MSG78 | 76.327 | 32.320 | -5.73 | -40.39 | 1.11 | 6.23 | 36.55 | -46.11 |
| MSG79 | 76.353 | 32.770 | -4.54 | -35.29 | 0.96 | 5.07 | 39.69 | -39.83 |
| MSG80 | 76.496 | 35.015 | 4.63 | -22.48 | 1.12 | 4.32 | 13.28 | -17.86 |
| MSG81 | 76.577 | 32.084 | -5.67 | -44.76 | 1.14 | 6.62 | 36.68 | -50.44 |
| MSG82 | 76.591 | 32.309 | -5.03 | -46.36 | 1.13 | 6.35 | 38.11 | -51.39 |
| MSG83 | 76.605 | 32.533 | -4.07 | -45.50 | 1.08 | 5.92 | 40.26 | -49.57 |
| MSG84 | 76.619 | 32.758 | -3.22 | -40.78 | 0.97 | 5.25 | 42.39 | -44.00 |

**References**

1. Wessel, P., Luis, J. F., Uieda, L., Scharroo, R., Wobbe, F., Smith, W. H. F., & Tian, D. The Generic Mapping Tools version 6. *Geochemistry, Geophysics, Geosystems*, **20**, 5556-5564. <https://doi.org/10.1029/2019GC008515> (2019).
2. Bendick, R. & Bilham, R. How perfect is the Himalayan arc?. *Geology* **29(9)**, 791-794. [https://doi.org/10.1130/0091-7613(2001)029<0791:HPITHA>2.0.CO;2](https://doi.org/10.1130/0091-7613(2001)029%3c0791:HPITHA%3e2.0.CO;2) (2001).
3. Banerjee, P., Bürgmann, R., Nagarajan, B. & Apel, E. Intraplate deformation of the Indian subcontinent. *Geophys. Res. Lett.* **35**, L18301. <https://doi.org/10.1029/2008GL035468> (2008).
4. Schiffman, C., Bali, B. S., Szeliga, W. & Bilham, R. Seismic slip deficit in the Kashmir Himalaya from GPS observations. *Geophys. Res. Lett.* **40**, 5642-5645. <https://doi.org/10.1002/2013GL057700> (2013).
5. Kundu, B., Yadav, R. K., Bali, B. S., Chowdhury, S. & Gahalaut, V. K. Oblique convergence and slip partitioning in the NW Himalaya: Implications from GPS measurements. *Tectonics* **33**, 2013-2024. <https://doi.org/10.1002/2014TC003633> (2014).
6. Jouanne, F. *et al.* Seismic coupling quantified on inferred decollements beneath the western syntaxis of the Himalaya. *Tectonics.* **39**, 1-20. <https://doi.org/10.1029/2020TC006122> (2020).
